# The Impact of Study Size on COVID‐19 Treatment Outcomes: A Meta‐Epidemiological Study Comparing Large and Small Randomized Controlled Trials: A Systematic Review and Meta‐Analyses

**DOI:** 10.1002/rmv.70125

**Published:** 2026-03-07

**Authors:** Dong Hyun Kim, Soojin Lim, Michael Eisenhut, Andreas Kronbichler, Eunyoung Kim, Min Seo Kim, Stefania I. Papatheodorou, Justin Stebbing, Yonghong Peng, Sarah Soyeon Oh, Jae Il Shin, Lee Smith

**Affiliations:** ^1^ Yonsei University College of Medicine Seoul Korea; ^2^ Luton & Dunstable University Hospital Bedfordshire Hospitals NHS Foundation Trust Luton UK; ^3^ Department of Internal Medicine IV Nephrology and Hypertension Medical University Innsbruck Innsbruck Austria; ^4^ Department of Health Medicine and Caring Sciences Linköping University Linköping Sweden; ^5^ Data Science Evidence‐Based and Clinical Research Laboratory Department of Health Social and Clinical Pharmacy College of Pharmacy Chung‐Ang University Seoul Korea; ^6^ Cardiovascular Disease Initiative Broad Institute of MIT and Harvard Cambridge Massachusetts USA; ^7^ Cardiovascular Research Center Massachusetts General Hospital Boston Massachusetts USA; ^8^ Department of Epidemiology Harvard T.H. Chan School of Public Health Boston Massachusetts USA; ^9^ Department of Biostatistics and Epidemiology Rutgers University Newark New Jersey USA; ^10^ School of Life Sciences Anglia Ruskin University Cambridge UK; ^11^ Faculty of Science and Engineering Anglia Ruskin University Cambridge UK; ^12^ Institute for Global Engagement & Empowerment Yonsei University Seoul Korea; ^13^ Department of Pediatrics Yonsei University College of Medicine Seoul Korea; ^14^ Severance Underwood Meta‐Research Center Institute of Convergence Science Yonsei University Seoul Korea; ^15^ Centre for Health Performance and Wellbeing Anglia Ruskin University Cambridge UK

**Keywords:** bias, COVID‐19, meta‐epidemiology, randomized controlled trials, small‐study effects, treatment outcome

## Abstract

Small randomized controlled trials (RCTs) in COVID‐19 meta‐analyses have been associated with more favourable treatment effects and reduced result stability. This study assessed how trial size impacts effect estimates, statistical stability, and risk of bias. Following PRISMA guidelines, we identified meta‐analyses of COVID‐19 treatments included in WHO, NIH, and the LIVING Project. Trials were classified by log‐scale sample size, and separate pooled meta‐analyses were conducted for large‐only, small‐only, and combined trials. Comparative metrics included the Ratio of Odds Ratios (ROR), Kappa statistics, Fragility Index (FI), Reverse Fragility Index (RFI), and Cochrane Risk of Bias assessments. Sensitivity analyses applied alternative size thresholds (≥ 1000 participants and median‐based cutoffs) and stratified results by treatment and outcome type. Across 25 meta‐analyses including 221 RCTs (46 large, 175 small), small trials produced more extreme estimates in 19 analyses and wider confidence intervals in 23. The pooled ROR was 0.85 (95% CI: 0.76–0.95; *P* = 0.004), decreasing to 0.81 (95% CI: 0.68–0.95; *P* = 0.011) when limited to small trials published before the first large trial. RORs remained below 1 across treatment and outcome types. Agreement between small and large trials was minimal, while large trials showed substantial agreement with overall estimates. Stability and bias profiles favoured large trials (FI: 14.0 vs. 4.0; RFI: 10.0 vs. 5.0). In conclusion, small RCTs tend to overestimate treatment effects and yield less precise, less stable results. Meta‐analyses should prioritise large, high‐quality trials and interpret small‐study findings with caution, particularly in rapidly evolving research contexts.

**Abbreviations**
CCP: COVID‐19 Convalescent Plasma
CI: Confidence Interval
COVID‐19: Coronavirus Disease 2019
FDA: Food and Drug Administration
FI: Fragility Index
IL: Interleukin
IQR: Interquartile Range
JAK: Janus Kinase
NA: Not Applicable
NIH: National Institutes of Health
NS: Not Significant
OR: Odds Ratio
P: P‐value
PRISMA: Preferred Reporting Items for Systematic Reviews and Meta‐Analyses
RCT: Randomized Controlled Trial
RFI: Reverse Fragility Index
RoB: Risk of Bias
ROR: Ratio of Odds Ratios
SARS‐CoV‐2: Severe Acute Respiratory Syndrome Coronavirus‐2

## 1 Introduction

The Coronavirus Disease‐2019 (COVID‐19) pandemic led to an unprecedented surge in clinical trials investigating a wide range of treatments, many of which have been synthesised through meta‐analyses to guide clinical practice [1]. As of 2024, meta‐analyses continue to be conducted to refine and update treatment guidelines [2, 3, 4, 5, 6, 7]. However, a critical issue in these meta‐analyses is the significant variation in size of the individual trials included, ranging from small trials with a limited number of participants to large‐scale randomized controlled trials (RCTs). The pandemic has underscored not only the importance of well‐designed randomized clinical trials but also the necessity for large‐scale trials that are structured according to a predefined and statistically planned protocol [8].

Small‐scale trials of hydroxychloroquine, for example, suggested potential benefits, leading to its widespread use despite insufficient evidence. This rapid adoption resulted in drug shortages for those who needed it for chronic conditions, and this was later contradicted by larger trials that found no benefit and potential harm [9]. Similarly, ivermectin gained attention from small early studies suggesting potential efficacy [10]. Social media and advocacy groups promoted it as a cure, despite weak evidence [11]. This led to widespread use in some areas, even when the Food and Drug Administration (FDA) advised against its use outside of clinical trials [12]. Larger trials, including a Cochrane systematic review, later showed no significant benefit and reported potential harm [13, 14]. These examples highlight the risks of prematurely relying on small trials for critical health decisions without the support of thorough, large‐scale research.

Previous research across medical fields [15, 16] has shown that small trials are more susceptible to publication bias. Studies that do not show significant differences between treatment arms may be rejected by journal editors and peer reviewers for being underpowered while those with significant results are more likely to be published. This phenomenon, known as ‘small‐study effects’, may lead to unstable meta‐analysis results [17]. These issues are particularly concerning in the context of COVID‐19, where the urgency of treatment development led to the inclusion of smaller, less rigorous trials in meta‐analyses, raising concerns about the reliability of clinical decisions and guidelines that heavily rely on these estimates [18]. Despite these concerns, there has been little exploration into the impact of study size on outcomes of COVID‐19 treatment meta‐analyses.

To address this gap, we conducted a comprehensive meta‐epidemiological analysis comparing the results of large and small RCTs within COVID‐19 treatment meta‐analyses. We selected a diverse range of treatments in accordance with the latest World Health Organisation (WHO) and National Institutes of Health (NIH) guidelines, as well as the LIVING Project, categorising trials as either large or small. By reanalysing these meta‐analyses separately for large and small trials, we assessed whether small‐study effects persist even within RCT‐only datasets—focussing on effect size, statistical stability, and risk of bias—to identify specific limitations of small trials and inform methodological improvements in future public health responses.

## 2 Methods

This meta‐epidemiological study examined the impact of trial size on treatment effect estimates in COVID‐19 meta‐analyses. As the analysis synthesised published meta‐analyses rather than primary studies, prospective registration was not undertaken. Such registration has limited applicability to meta‐epidemiological research [19, 20, 21], particularly in the context of rapidly evolving COVID‐19 evidence. Nevertheless, all methodological criteria were defined a priori and applied consistently. The study was conducted in accordance with the Preferred Reporting Items for Systematic Reviews and Meta‐Analyses (PRISMA) guidelines.

### 2.1 Search Strategy and Study Selection

A systematic search of PubMed and the Cochrane Database of Systematic Reviews was performed on February 6, 2025, to identify meta‐analyses of COVID‐19 treatments referenced in WHO and NIH guidelines, as well as the LIVING Project [22, 23, 24]. For each treatment, we prioritised the most recent meta‐analysis that included the largest number of trials, particularly those assessing all‐cause mortality as a primary or secondary outcome. Eligible meta‐analyses included only RCTs with non‐zero events and a Cochrane Risk of Bias (RoB) assessment, which was carefully reviewed and supplemented by all authors.

Treatments were categorised into four groups according to their primary mechanisms of action in COVID‐19 trials. Colchicine was classified as miscellaneous due to its dual action in microtubule inhibition and potential antiviral effects [25, 26]. The search terms and superseded studies are outlined in Supporting Information S1: Appendix 1.

1. Anti‐infective agents: Remdesivir [27], Molnupiravir [28], Ivermectin [14], Lopinavir [29], Hydroxychloroquine [29], Azithromycin [30], Favipiravir [31].
2. Anti‐inflammatory and immunomodulatory agents: Corticosteroids, [32] IL‐6 (Interleukin‐6) Receptor Blockers (Tocilizumab, Sarilumab), [33] Janus Kinase (JAK) Inhibitors [34], Interferon [35], IL‐1 Receptor Blockers (Anakinra, Canakinumab) [36].
3. Anti‐Severe Acute Respiratory Syndrome Coronavirus‐2 (SARS‐CoV‐2) monoclonal antibodies (Casirivimab‐Imdevimab) [37].
4. Miscellaneous agents: Fluvoxamine [38], Colchicine [39], COVID‐19 Convalescent Plasma (CCP) [40].

### 2.2 Trial Size Classification

To avoid misclassification arising from fixed thresholds or quantile‐based approaches [19, 20, 21], trial size was defined within each treatment‐specific meta‐analysis. Large trials were those whose base‐10 logarithms (rounded down) of treatment, control, and total sample sizes matched those of the largest trial in the same meta‐analysis. This approach minimised arbitrary dichotomisation and accounted for skewed size distributions commonly observed in COVID‐19 trials.

In meta‐analyses with highly skewed size distributions, empirical adjustments were made to ensure meaningful separation—for instance, allowing more trials to be classified as large when a single exceptionally large study dominated the evidence base (e.g., CCP) [40], or fewer when trial sizes were relatively homogeneous (e.g., fluvoxamine) [38]. All classifications were determined within individual meta‐analyses, agreed upon by all authors, and applied consistently across all analyses, as detailed in online Supporting Information S1: Appendix 1. Sensitivity analyses using traditional cutoffs (total participant cutoff of 1000 participants, median splits) were conducted to test robustness.

### 2.3 Meta‐Analytic Structure

For each treatment, three random‐effects meta‐analyses were conducted using the DerSimonian‐Laird model [41]:

1. Large Trials Only: Pooled meta‐analysis including only large trials.
2. Small Trials Only: Pooled meta‐analysis including only small trials.
3. All Trials (Combined): Pooled meta‐analysis including all trials regardless of size.

Effect sizes were reported as odds ratios (ORs), with outcomes reversed as needed so that ORs < 1 indicated benefit. Between‐trial heterogeneity was assessed using τ^2^, Cochran's Q, and I^2^ statistics.

### 2.4 Primary Outcome: Small‐Study Effect

The Ratio of Odds Ratios (ROR) [21, 42]—calculated by dividing the pooled OR of small trials by that of large trials— was used to evaluate whether treatment effects estimated from small trials differed systematically from those derived from large trials. An ROR less than 1 indicates more favourable effect estimates in small trials.

We performed an additional analysis by restricting the small trials to those published on or before the year of the first large trial. This step was taken to examine whether there was a diminishing effect in later smaller trials compared to earlier small trials, consistent with previous research [43, 44, 45, 46]. Leave‐one‐out sensitivity analysis and subgroup analyses stratified by treatment class and outcome type were performed.

To quantify the agreement between the results of the large, small, and combined analyses, we calculated the Kappa statistic [47] for each set of comparisons. The outcomes of the meta‐analyses were classified as either positive (OR< 1) or negative (OR≥ 1). We interpreted Kappa values using Cohen's standard guidelines [48].

### 2.5 Secondary Outcome: Stability and Risk of Bias

We assessed the stability of trial results using the Fragility Index (FI) [49, 50] and Reverse Fragility Index (RFI) [51]. The FI represents the number of event changes required to render a statistically significant result non‐significant, while the RFI applies to non‐significant results and indicates how many event changes would be needed to achieve significance. Higher values in both indicate greater result stability. Given the absence of a standardized framework for applying FI/RFI at the meta‐analytic level, these metrics were calculated for individual trials and used to compare overall stability patterns between large and small trials. Group‐level differences were assessed using the Mann–Whitney *U* test.

RoB assessments from the selected meta‐analyses were carefully reviewed by authors and corrected or supplemented as needed according to the original Cochrane RoB 1.0 or 2.0 tool, as detailed in Supporting Information S1: Appendix 1. Results were aggregated by tool version and trial size, and trend *p*‐values from Wilcoxon rank‐sum tests were used to compare the proportion of high‐risk domains between large and small trials within each RoB tool.

### 2.6 Statistical Analyses

All statistical analyses were conducted using R package, version 4.4.1. Data analysis was conducted from February to May 2025. The threshold for significance was set at a 2‐tailed *p*‐value below 0.05 and descriptive data were expressed as either “Median (interquartile range [IQR])” or “No. (%)”.

## 3 Results

### 3.1 Treatment and Study Selection

**FIGURE 1:**
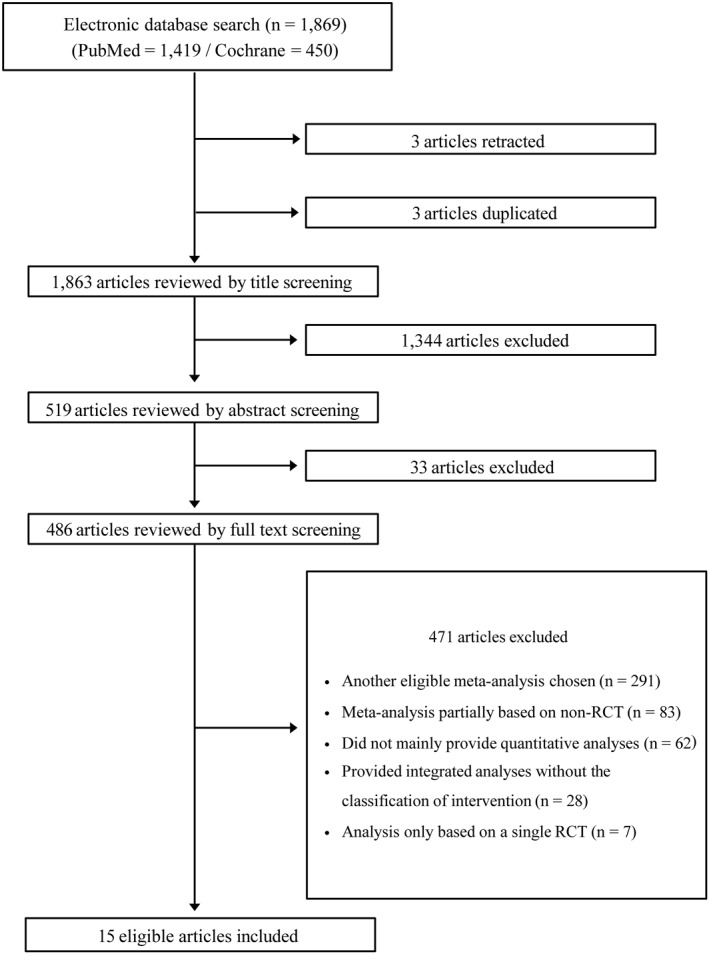
PRISMA flow diagram. PRISMA, Preferred Reporting Items for Systematic Reviews and Meta‐Analyses; RCT, Randomized Controlled Trial.

The database search identified 1869 records (1419 from PubMed and 450 from Cochrane). After removing duplicates (*n* = 3) and retracted publications (*n* = 3), 1344 articles were excluded at the title level and 33 at the abstract level. Of 486 articles assessed in full text, 471 were excluded because they were superseded (*n* = 291), included non‐RCTs (*n* = 83), lacked quantitative analyses (*n* = 62), provided integrated analyses without intervention stratification (*n* = 28), or were based on a single RCT (*n* = 7). Ultimately, 15 articles met eligibility criteria, contributing 25 meta‐analyses. The PRISMA flow diagram and checklist are shown in Figure 1 and Supporting Information S1: Appendix 2.

### 3.2 Characteristics of Collected RCTs

The 25 meta‐analyses encompassed 221 RCTs evaluating four outcomes: all‐cause mortality (21 meta‐analyses, 204 trials), progression to clinical symptoms (2 meta‐analyses, 4 trials), need for mechanical ventilation (1 meta‐analysis, 5 trials), and clinical improvement (1 meta‐analysis, 8 trials). 46 trials were classified as large [median study size: 1374 (IQR: 523.50–4057.25)], with five of these trials showing statistically significant results. In contrast, 175 trials were classified as small [median study size: 126 (IQR: 61.0–292.5)], with 15 showing statistically significant results. 112 small trials were published before or up to the year of the first large trial. Individual study characteristics such as range of participants and *p*‐value are provided in Supporting Information S1: Table 1.

### 3.3 Investigation of Small‐Study Effects

**FIGURE 2:**
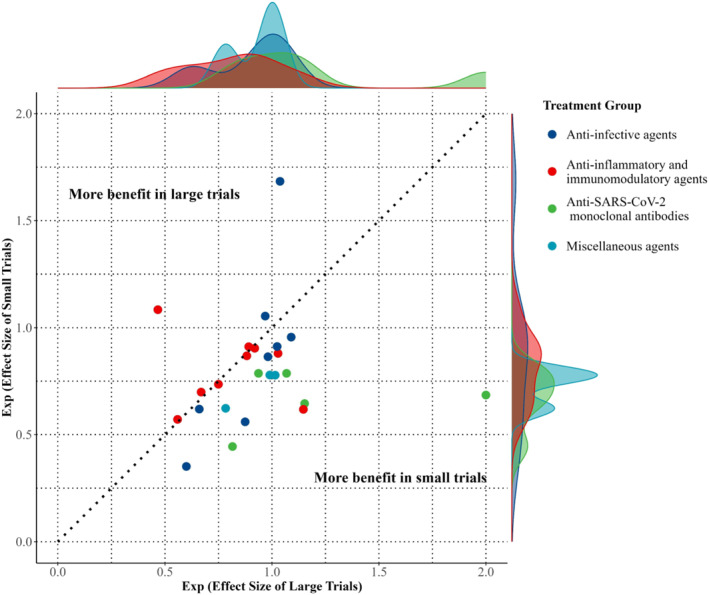
Scatter plot of effect size comparisons between large and small trials. Each point represents a meta‐analysis, comparing the effect size from large trials (x‐axis) and small trials (y‐axis). Points are coloured by treatment group: anti‐infective agents (blue), anti‐inflammatory and immunomodulatory agents (red), anti‐SARS‐CoV‐2 monoclonal antibodies (green), and miscellaneous agents (cyan). The dotted diagonal line represents equal effect estimates from large and small trials. Points above the line indicate a greater benefit in large trials, while points below the line indicate a greater benefit in small trials. The marginal density plots illustrate the distribution of effect sizes by trial size and treatment group. Exp, Exponential.

As shown in Table 1 and Figure 2, small trials yielded more extreme effect estimates in 19 of 25 meta‐analyses and had wider 95% confidence intervals than large trials in 23 of 25, indicating less precision and greater variability. The pooled ROR was 0.85 (95% CI: 0.76–0.95; *P* = 0.004), demonstrating a statistically significant association between smaller trial size and more favourable treatment effect estimates compared with large trials, as shown in Figure 3A. Supporting Information S1: Figure 1 presents the forest plots of small‐only, large‐only, and combined ORs for each meta‐analysis.

**TABLE 1:** Pooled estimates of large trials, small trials, and overall.

Treatment	Outcome		Large trials					Small trials						Overall			
		No.	Treatment group	Control group	Random effects (95% CI)	*p*	Range of participants	No.	Treatment group	Control group	Random effects (95% CI)	*p*	Range of participants	Random effects (95% CI)	*p*	I^2^ (%)	*P*_*comp*_
A. Pooled estimates of anti‐infective agents																	
Remdesivir [27]	Mortality	1	301/2743	303/2708	0.98 (0.84–1.14)	0.800	5451	6	132/1432	139/1352	0.86 (0.69–1.09)	0.208	70–1062	0.94 (0.81–1.08)	0.380	0	0.363
Molnupiravir [28]	Mortality	1	3/12529	5/12525	0.60 (0.14–2.51)	0.484	25054	4	14/2676	12/2251	0.35 (0.05–2.61)	0.307	107–1408	0.43 (0.10–1.82)	0.490	51	0.670
Ivermectin [14]	Mortality	1	21/679	24/679	0.88 (0.49–1.56)	0.650	1358	3	7/761	14/741	0.56 (0.18–1.78)	0.326	24–501	0.75 (0.43–1.30)	0.309	0	0.499
Lopinavir [29]	Mortality	2	522/3027	913/4804	1.02 (0.93–1.13)	0.623	2791–5040	3	35/522	38/492	0.91 (0.60–1.39)	0.668	51–471	1.02 (0.93–1.12)	0.702	0	0.597
Hydroxy‐chloroquine [29]	Mortality	2	525/2508	874/4061	1.09 (0.99–1.20)	0.077	1853–4716	16	131/1979	142/2009	0.96 (0.78–1.18)	0.672	2–479	1.07 (0.98–1.16)	0.152	0	0.264
Azithromycin [30]	Mortality^a^	1	561/2582	1162/5181	0.97 (0.89–1.06)	0.490	7763	1	90/214	73/183	1.05 (0.83–1.34)	0.663	397	0.98 (0.90–1.06)	0.616	0	0.513
	Mortality^b^	1	5/172	7/159	0.66 (0.21–2.04)	0.470	331	2	1/132	2/131	0.62 (0.08–4.97)	0.652	111–152	0.65 (0.24–1.75)	0.396	0	0.958
Favipiravir [31]	Mortality	3	45/853	45/802	1.04 (0.58–1.85)	0.901	156–500	4	4/228	1/191	1.68 (0.35–8.03)	0.513	50–148	1.09 (0.64–1.84)	0.758	0	0.568
B. Pooled estimates of anti‐inflammatory and immunomodulatory agents																	
Cortico‐steroids [32]	Mortality	1	482/2104	1110/4321	**0.89 (0.81–0.98)**	**0.016**	6425	11	250/1916	272/2140	0.91 (0.80–1.04)	0.153	30–1856	**0.90 (0.83–0.97)**	**0.005**	19	0.791
IL‐6 Receptor blockers [33]	Mortality^c^ ^,^ ^d^	1	621/2022	729/2094	**0.88 (0.81–0.96)**	**0.005**	4116	17	284/1858	283/1480	0.87 (0.74–1.02)	0.092	21–778	**0.88 (0.81–0.94)**	**0.001**	0	0.866
	Mortality^c^ ^,^ ^e^	1	323/972	151/418	0.92 (0.79–1.07)	0.293	1390	8	149/880	97/531	0.90 (0.72–1.13)	0.375	21–452	0.91 (0.80–1.04)	0.171	0	0.899
	Clinical improve‐ment^f^	1	473/1044	126/286	1.03 (0.89–1.19)	0.708	1330	7	229/703	116/392	0.88 (0.74–1.05)	0.147	30–457	0.96 (0.85–1.08)	0.479	0	0.178
JAK inhibitors [34]	Mortality	3	113/1795	167/1773	**0.67 (0.53–0.84)**	**0.001**	1010–1525	2	34/190	47/186	**0.70 (0.49–0.99)**	**0.046**	101–275	**0.68 (0.56–0.82)**	**< 0.001**	0	0.837
Interferon [35]	Mortality	2	252/1971	219/1974	1.15 (0.97–1.36)	0.111	817–3128	4	33/189	42/163	0.62 (0.36–1.07)	0.087	40–105	0.84 (0.56–1.25)	0.382	59	**0.036**
IL‐1 Receptor blockers [36]	Mortality^g^	1	13/405	13/189	**0.47 (0.22–0.99)**	**0.046**	594	4	34/155	32/178	1.08 (0.70–1.67)	0.715	30–118	0.92 (0.56–1.51)	0.738	43	0.056
	Mortality^h^	1	12/227	16/227	0.75 (0.36–1.55)	0.437	454	1	4/29	3/16	0.74 (0.19–2.89)	0.660	45	0.75 (0.39–1.42)	0.372	0	0.980
	Risk of requiring mechanical ventilation	2	23/632	29/413	**0.56 (0.33–0.96)**	**0.034**	451–594	3	8/109	14/102	0.57 (0.16–2.04)	0.388	30–114	**0.55 (0.35–0.86)**	**0.009**	0	0.975
C. Pooled estimates of anti‐SARS‐CoV‐2 monoclonal antibodies																	
Anti‐SARS‐CoV‐2 monoclonal antibodies [37]	Mortality^i^	1	396/1633	452/1520	**0.82 (0.73–0.92)**	**0.001**	3153	1	24/360	24/160	**0.44 (0.26–0.76)**	**0.003**	520	0.64 (0.36–1.14)	0.132	79	**0.030**
	Mortality^j^	1	410/2636	384/2636	1.07 (0.94–1.21)	0.317	5272	1	26/369	18/201	0.79 (0.44–1.40)	0.414	570	1.05 (0.91–1.21)	0.514	3	0.311
	Mortality^k^	1	943/4839	1029/4946	0.94 (0.87–1.01)	0.105	9785	3	413/2895	484/2482	**0.79 (0.70–0.89)**	**< 0.001**	1197–2696	**0.83 (0.73–0.95)**	**0.008**	60	**0.017**
	Progression to present clinical symptoms^i^	1	70/842	13/841	**5.38 (3.00‐9.65)**	**< 0.001**	1683	1	29/100	44/104	0.69 (0.47–1.00)	0.051	204	1.90 (0.25–14.28)	0.534	97	**< 0.001**
	Progression to present clinical symptoms^k^	1	14/729	4/240	1.15 (0.38–3.47)	0.801	969	1	34/155	53/156	**0.65 (0.45–0.93)**	**0.029**	311	**0.68 (0.48–0.97)**	**0.034**	0	0.328
D. Pooled estimates of miscellaneous agents																	
Fluvoxamine [38]	Mortality^l^	1	76/741	99/756	0.78 (0.59–1.04)	0.089	1497	4	10/1611	18/1540	0.62 (0.29–1.35)	0.232	152–1277	**0.76 (0.59–0.99)**	**0.045**	0	0.585
Colchicine [39]	Mortality	4	1454/11088	1459/11232	1.01 (0.95–1.08)	0.658	2611–11340	16	188/1775	237/1729	**0.78 (0.61–0.99)**	**0.038**	30–1279	0.99 (0.93–1.05)	0.778	0	**0.034**
CCP [40]	Mortality	9	2197/9390	2044/8616	0.99 (0.94–1.04)	0.701	333–11558	29	351/1876	338/1593	**0.78 (0.65–0.93)**	**0.007**	29–400	0.97 (0.92–1.01)	0.172	33	**0.013**

*Note:* The P_comp_ column shows the *p*‐value for the Ratio of Odds Ratios (ROR) difference. Results with statistical significance were marked bold.

Abbreviations: OR, Odds Ratio; CI, Confidence Interval; IL, Interleukin; JAK, Janus Kinase; SARS‐CoV‐2, Severe Acute Respiratory Syndrome Coronavirus‐2; Comp, Comparison.

^a^ Severe patients.

^b^ Non‐severe patients.

^c^ Tocilizumab.

^d^ All‐cause mortality at day 28.

^e^ All‐cause mortality at day 60.

^f^ Sarilumab.

^g^ Anakinra.

^h^ Canakinumab.

^i^ Seronegative baseline patients.

^j^ Seropositive baseline patients.

^k^ Overall baseline patients.

^l^ Inpatients.

FIGURE 3Forest plot of pooled meta‐analysis of ROR (A) Between large and small trials (B) Between large and small trials preceding the large trials. The blue square shows the pooled ROR, while white and black dots represent individual meta‐analyses' ROR, with black dots indicating significance. The arrow indicates when the effect size interval exceeds the range of 0.1–10. CI, Confidence Interval; IL, Interleukin; JAK, Janus Kinase; ROR, Ratio of Odds Ratio. Footnotes: ^a^ Severe patients, ^b^ Non‐severe patients, ^c^ Tocilizumab, ^d^ All‐cause mortality at day 28, ^e^ All‐cause mortality at day 60, ^f^ Sarilumab, ^g^ Anakinra, ^h^ Canakinumab, ^i^ Seronegative baseline patients, ^j^ Seropositive baseline patients, ^k^ Overall baseline patients, ^l^ Inpatients.

**Figure:**
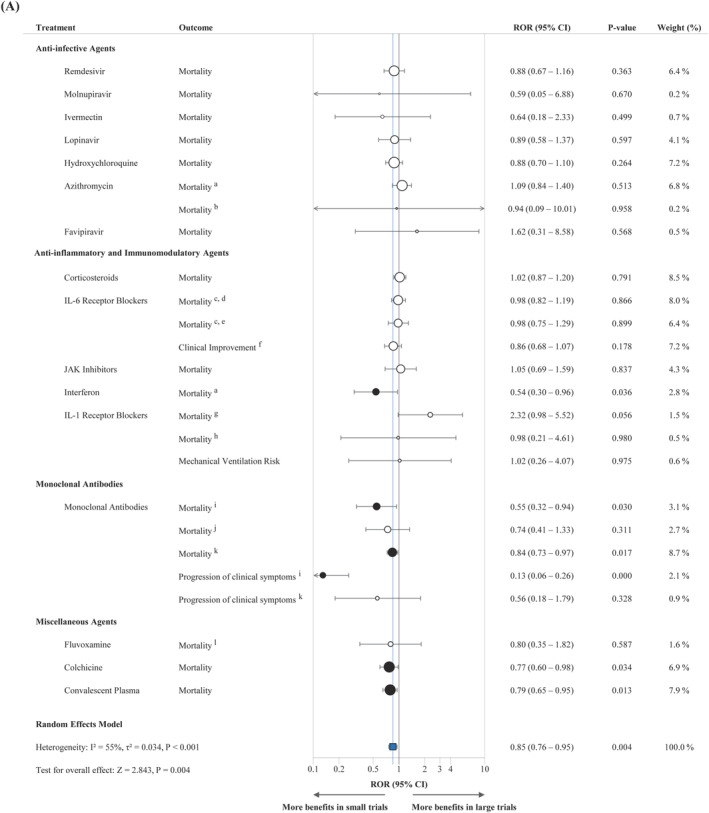

**Figure:**
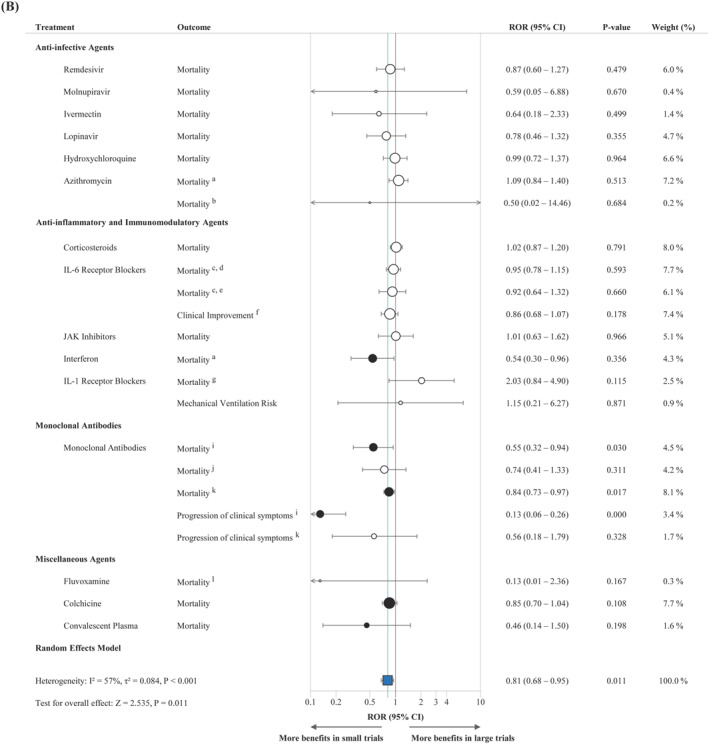

Agreement between large and small trials was minimal (*κ* = −0.02; *P* = 0.918), as was agreement between small trials and pooled estimates (*κ* = 0.12; *P* = 0.538). In contrast, agreement between large trials and pooled estimates was substantial and statistically significant (*κ* = 0.62; *P* = 0.001). Using a stricter definition of positivity (OR < 1 with statistical significance) yielded similar results, with low agreement for large versus small trials (*κ* = 0.12, *P* = 0.539) and small versus pooled estimates (*κ* = 0.27, *P* = 0.169), and persistent moderate agreement between large trials and pooled estimates (*κ* = 0.48, *P* = 0.016).

Limiting small studies to those published before or up to the first large trial decreased the pooled ROR to 0.81 (95% CI: 0.68–0.95; *P* = 0.011), as shown in Figure 3B. Direct comparison of pre‐ and post‐large trial estimates showed that 19 of 23 meta‐analyses favoured more pronounced effects before large trials emerged, supported by a pooled ROR of 0.79 (95% CI: 0.68–0.93; *P* = 0.005) (Supporting Information S1: Figure 2). Two meta‐analyses (favipiravir and canakinumab for all‐cause mortality) were excluded from this comparison because all small trials were published after the first large trial.

### 3.4 Subgroup and Sensitivity Analyses

In all treatment groups, the pooled ROR remained below 1, indicating a consistent pattern of smaller trials showing more favourable treatment effects. Specifically, the pooled ROR was 0.93 (95% CI: 0.82–1.07; *P* = 0.329) for anti‐infective agents, 0.97 (95% CI: 0.88–1.07; *P* = 0.535) for anti‐inflammatory and immunomodulatory agents, 0.49 (95% CI: 0.25–0.96; *P* = 0.037) for anti‐SARS‐CoV‐2 monoclonal antibodies, and 0.78 (95% CI: 0.67–0.90; *P* < 0.001) for miscellaneous agents. Subgroup analyses by outcome type showed that the pooled ROR for all‐cause mortality was 0.90 (95% CI: 0.83–0.97; *P* = 0.004), indicating significantly more favourable effects in small trials. For 4 meta‐analyses with non‐mortality outcomes, the pooled ROR was 0.48 (95% CI: 0.18–1.28; *P* = 0.143), suggesting a similar trend, although not statistically significant due to limited data.

Leave‐one‐out analyses yielded stable pooled RORs ranging from 0.83 to 0.89. The heterogeneity investigation showed that excluding 6 meta‐analyses with only one large and one small trial reduced heterogeneity to I^2^ = 0.0, while the pooled ROR remained significant and consistent (0.89; 95% CI: 0.83–0.96; *P* = 0.001), as detailed in Supporting Information S1: Appendix 3. Alternative trial size cutoffs, including a fixed threshold of 1000 participants and a median split, as well as alternative random‐effects estimators, yielded similar pooled ROR estimates (Supporting Information S1: Appendix 4).

### 3.5 Comparisons of Stability and Bias Between Large and Small Trials

**FIGURE 4:**
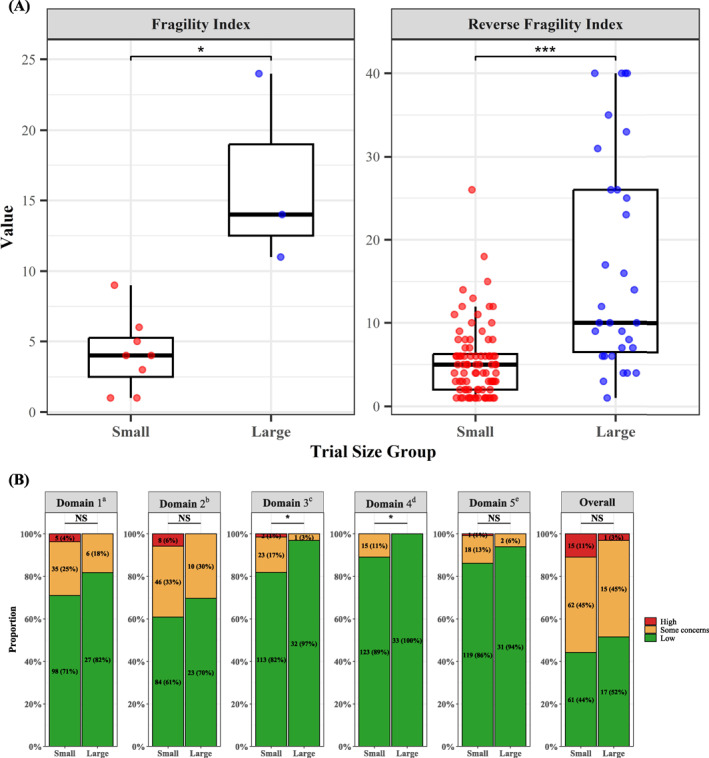
Stability and bias profiles of large and small trials (A) Distributions of FI and RFI in large and small trials. (B) Distribution of RoB 2.0 Assessments in large and small trials. FI, Fragility Index; NS, Not Significant; RFI, Reverse Fragility Index. Footnotes: ^a^ Bias arising from the randomisation process, ^b^ Bias due to deviations from intended intervention, ^c^ Bias due to missing outcome data, ^d^ Bias in measurement of the outcome, ^e^ Bias in selection of the reported result.

Among meta‐analyses with statistically significant results, large trials exhibited higher stability than small trials, with median FI values of 14.0 (IQR: 12.5–19.0) versus 4.0 (IQR: 2.5–5.3), respectively (*P* = 0.018). Similarly, among non‐significant results, large trials showed higher median RFI values than small trials [10.0 (IQR, 6.5–26.0) versus 5.0 (IQR, 2.0–6.3); *P* < 0.001], as shown in Figure 4A.

Of the included meta‐analyses, 10 used the Cochrane RoB 1.0 tool and 15 used RoB 2.0. Given the limited number of large trials assessed with RoB 1.0, comparative analyses focused on RoB 2.0. Large trials consistently demonstrated a higher proportion of low‐risk assessments and fewer high‐risk domains than small trials, with significant differences observed for missing outcome data (*P* = 0.031) and outcome measurement (*P* = 0.048), as illustrated in Figure 4B. Summary data are provided in Supporting Information S1: Table 2, with full trial‐level assessments in Supporting Information S1: Table 3. Results were consistent across alternative trial size definitions (Supporting Information S1: appendix 4, Supporting Information S1: Figure 3).

## 4 Discussion

### 4.1 Summary

In this meta‐epidemiological study of COVID‐19 treatment meta‐analyses, small trials consistently produced more favourable and less precise effect estimates than large trials. Small trials showed wider confidence intervals in most comparisons and more extreme effect estimates in nearly three‐quarters of meta‐analyses. These differences translated into a pooled ROR of 0.85, indicating systematic overestimation of treatment effects in smaller trials. The finding remained robust across extensive sensitivity analyses, alternative size thresholds, and subgroup stratifications by treatment class and outcome type.

When restricted to small trials published before the first large trial, the pooled ROR decreased to 0.81, indicating greater overestimation of treatment effects in early evidence. Agreement analyses demonstrated minimal concordance between small and large trials, whereas pooled estimates aligned substantially with large trials. Complementary stability metrics showed that large trials were markedly more robust, with higher FI and RFI values, and RoB assessments revealed greater susceptibility to bias among small trials, particularly related to missing outcome data and outcome measurement.

### 4.2 Significance of This Study

Although small‐study effects have been well documented in other areas of medicine, their role within COVID‐19 treatment meta‐analyses has not been systematically examined. While prior meta‐epidemiological studies in the COVID‐19 literature have primarily focused on methodological quality or publication venue [52, 53], none have directly quantified small‐study bias across treatment domains. This study extends established frameworks to the COVID‐19 setting and demonstrates that small‐study effects persist even within RCT‐only datasets.

Notably, conventional funnel‐plot‐based methods detected asymmetry in only two cases, consistent with prior reports of limited sensitivity in heterogeneous and rapidly evolving evidence bases [54]. In contrast, the ROR approach revealed an approximately 15% overestimation attributable to small trials, likely reflecting the proliferation of underpowered trials early in the pandemic. Importantly, this magnitude is comparable to the mortality reduction later confirmed for corticosteroids in large trials [55], illustrating how small‐study bias alone can mimic the apparent effect size of an effective therapy. However, as our pre‐versus post‐large analysis confirms, the prompt arrival of large, well‐conducted platform trials attenuated this bias over time, preventing it from reaching historical extremes [56].

FI, RFI, and structured RoB assessments, further reinforce the conclusion that larger trials confer greater evidentiary stability. Notably, smaller trials showed higher risk of missing data and outcome misclassification, likely reflecting limited follow‐up infrastructure, with implications for internal validity and meta‐analytic accuracy [57, 58]. However, given that these metrics are restricted to binary outcomes and no standardized meta‐analytic framework currently exists, they should be regarded as complementary measures of robustness rather than replacements for effect estimates and confidence intervals.

Despite these concerns, small trials remain indispensable in early outbreak phases due to their feasibility, speed, and lower resource requirements [59, 60]. The objective should not be exclusion, but rather the integration of such trials into meta‐analyses through standardized methodological safeguards. Random‐effects models address heterogeneity by weighting more precise studies, while advanced approaches may further mitigate bias, each with inherent limitations [61]. The trim‐and‐fill method [62] assumes distributional symmetry; the Copas selection model [63] is highly sensitive to model assumptions; regression‐based approaches [64] may oversimplify causal pathways; and trial sequential analysis [65] focuses on individual trial conclusiveness rather than systematic trends. Accordingly, our findings inform evidence synthesis and guideline development focused on evidentiary certainty, rather than prescribing sample‐size targets for individual trials.

Furthermore, restricting the analysis to smaller trials published before the publication of large trial showed a more pronounced small‐study effect. This finding aligns with recent research [66] comparing mega trials to earlier‐published smaller trials, raising a concern that smaller trials conducted after a large trial may adjust methodologies and reporting to align with the large trial's standards. Early large trials, such as the WHO Solidarity Trial [67]. therefore play a critical role in guiding subsequent research and preventing cycles of premature adoption and regulatory reversal, as seen with hydroxychloroquine. These dynamics are further amplified by social media, where fragile early findings can rapidly lead to inappropriate resource use and adverse patient outcomes [68, 69].

### 4.3 Limitations

This study has several limitations. First, our trial size classification relied on a flexible, within–meta‐analysis log‐scale approach that required case‐specific decisions, which may limit reproducibility, even though sensitivity analyses using alternative cutoffs yielded consistent results. Second, subgroup and sensitivity analyses were exploratory and should be interpreted cautiously given the limited number of meta‐analyses and potential multiplicity. Third, we prioritised the most comprehensive meta‐analysis per treatment to ensure analytic independence. While this avoided redundancy, it narrowed the evidence base and may have introduced selection bias. Additionally, the absence of prospective registration may limit transparency, although all analyses were predefined and systematically applied. Finally, restriction to RCTs enhanced internal validity but limited generalisability and power for certain treatments and outcomes with few large trials. Future work should employ more objective classification criteria, incorporate high‐quality observational studies, and expand the scope of treatments and outcomes to improve generalisability.

## 5 Conclusion

In conclusion, this study underscores the importance to prioritise large, high‐quality trials in meta‐analyses to ensure the accurate, reliable conclusions for clinical practice and public health policy. By addressing small‐study effects, it offers a crucial framework to strengthen future meta‐research. While large trials should be given priority, small trials still provide valuable early insights and require fewer resources. Their inclusion in meta‐analyses should be carefully managed with advanced statistical methods and standardized protocols to minimise bias and enhance overall reliability.

Supporting Information S1: files include detailed classification criteria, sensitivity analyses, and additional stability and risk‐of‐bias assessments. Supporting Information S1: figures and tables provide extended results supporting the main findings.

## Author Contributions

J.I.S. is the guarantor, assuming full responsibility for the integrity of the work and the study's conduct, with access to all data and authority over the decision to publish. All authors contributed to the study's concept and design, as well as the acquisition, analysis, and interpretation of data. The manuscript was draughted by all authors, with critical feedback and revisions provided by all for important intellectual content. D.K. and S.L. performed the statistical analysis. Supervision of the overall project was provided by J.I.S.

## Funding

This work was supported by the Yonsei Fellowship, funded by Lee Youn Jae.

## Ethics Statement

The authors have nothing to report.

## Consent

The authors have nothing to report.

## Conflicts of Interest

The authors have no competing interests to declare.

## Patient and Public Involvement

Patients and/or the public were not involved in the design, or conduct, or reporting, or dissemination plans of this research.

## Supporting information


Supporting Information S1

## Data Availability

The data that support the findings of this study are available in PubMed at https://pubmed.ncbi.nlm.nih.gov/.
