# Supplementary material for: The Impact of Study Size on COVID‐19 Treatment Outcomes: A Meta‐Epidemiological Study Comparing Large and Small Randomized Controlled Trials: A Systematic Review and Meta‐Analyses
Source: Rev Med Virol. 2026 Mar 7;36(2):e70125. doi: 10.1002/rmv.70125 (PMC12966952; doi:10.1002/rmv.70125)
Supplement: Supplementary file 1 — Supporting Information S1 [file RMV-36-e70125-s001.docx]

**Supplemental Appendix 1. Search strategy and RoB value standardization criteria**

**Supplemental Appendix 2. PRISMA checklist**

**Supplemental Appendix 3. Results of heterogeneity investigation**

**Supplemental Appendix 4. Sensitivity and subgroup analyses using alternative definitions of trial size and random-effects estimators**

**Supplemental Figure 1. Forest plot of meta-analysis by study size**

**Supplemental Figure 2. Scatter plot of effect size comparisons between pre-large and post-large trials**

**Supplemental Figure 3**. **Stability and bias profiles by trial size across alternative thresholds**

**(A)** **Distributions of FI and RFI in large and small trials (≥1,000-Participant Threshold)**

**(B)** **Distributions of FI and RFI in large and small trials (Median-Based Threshold)**

**(C)** **Distribution of RoB 2.0 Assessments in large and small trials (≥1,000-Participant Threshold)**

**(D)** **Distribution of RoB 2.0 Assessments in large and small trials** **(Median-Based Threshold)**

**Supplemental Table 1. Individual study statistics**

**(A) Individual study statistics of anti-infective agents**

**(B) Individual study statistics of anti-inflammatory and immunomodulatory agents**

**(C) Individual study statistics of anti-SARS-CoV-2 monoclonal antibodies**

**(D) Individual study statistics of miscellaneous agents**

**Supplemental Table 2. Comparisons of RoB between large and small trials**

**(A) Comparisons of RoB 1.0 between large and small trials**

**(B) Comparisons of RoB 2.0 between large and small trials**

**Supplemental Table 3. Individual RoB evaluation**

**(A) Studies evaluated by RoB 1.0 tool**

**(B) Studies evaluated by RoB 2.0 tool**

**Supplemental References**

**Supplemental Appendix 1. Search strategy and RoB value standardization criteria**

The systematic search of the PubMed and Cochrane databases was conducted on February 6^th^, 2025, to identify relevant meta-analyses of COVID-19 treatments. The search queries for the PubMed and Cochrane databases are provided below. For each treatment, we prioritized the latest and most comprehensive meta-analyses that focused on all-cause mortality, included sufficient trials with non-zero events, assessed Cochrane Risk of Bias, and comprised only RCTs. The selected treatments were then grouped into four main categories, based on their mechanisms of actions.

**Search queries**

1. PubMed: covid meta ((remdesivir OR VV116) OR molnupiravir OR ivermectin OR (lopinavir/ritonavir OR kaletra) OR (hydroxychloroquine OR chloroquine OR azithromycin) OR favipiravir OR corticosteroids OR (anakinra OR IL-1 Receptor Blockers) OR (interleukin OR tocilizumab OR actemra OR sarilumab OR IL-6 Receptor Blockers) OR (JAK OR Janus OR baricitinib OR ruxolitinib OR tofacitinib) OR interferon OR (casirivimab-imdevimab OR REGEN-COV OR antibodies) OR fluvoxamine OR colchicine OR (CCP OR convalescent plasma))
2. Cochrane: covid AND meta AND ((remdesivir OR VV116) OR molnupiravir OR ivermectin OR ((lopinavir AND ritonavir) OR kaletra) OR (hydroxychloroquine OR chloroquine OR azithromycin) OR favipiravir OR corticosteroids OR (anakinra OR IL-1 Receptor Blockers) OR (interleukin OR tocilizumab OR actemra OR sarilumab OR IL-6 Receptor Blockers) OR (JAK OR Janus OR baricitinib OR ruxolitinib OR tofacitinib) OR interferon OR (casirivimab-imdevimab OR REGEN-COV OR antibodies) OR fluvoxamine OR colchicine OR (CCP OR convalescent plasma))

**Treatment classification**

1. Anti-infective agents: Remdesivir,^1^ Molnupiravir,^2^ Ivermectin,^3^ Lopinavir,^4^ Hydroxychloroquine,^4^ Azithromycin,^5^ Favipiravir^6^
2. Anti-inflammatory and immunomodulatory agents: Corticosteroids,^7^ IL-6 (Interleukin-6) Receptor Blockers (Tocilizumab, Sarilumab),^8^ Janus Kinase (JAK) Inhibitors,^9^ Interferon,^10^ IL-1 Receptor Blockers (Anakinra, Canakinumab)^11^
3. Anti-Severe Acute Respiratory Syndrome Coronavirus-2 (SARS-CoV-2) monoclonal antibodies (Casirivimab-Imdevimab)^12^
4. Miscellaneous agents: Fluvoxamine,^13^ Colchicine,^14^ COVID-19 Convalescent Plasma (CCP)^15^

**Superseded papers**

For molnupiravir, two candidates^2,16^of similar eligibility were identified; the one including a comprehensive meta-analysis of all dosage types (200, 400, 800 mg) was selected. For anti-SARS-CoV-2 monoclonal antibodies, two candidates^12,17^ were also identified, but the one with fewer zero-events^12^, despite including fewer antibody types, was chosen. Lastly, umifenovir was initially considered, but due to the most eligible study^18^ having too many zero-events, the treatment was excluded.

**RoB value standardization criteria**

We standardized the Risk of Bias (RoB) evaluations for trials using RoB 1 and RoB 2 tools to ensure consistency. For RoB 1.0 tool, values that did not match the standard categories ('Low,' 'High,' 'Unclear') were reassigned based on equivalencies such as '+' for 'Low' and '-' for 'High.' Similarly, for RoB 2.0 tool, the standardization process adjusted values according to pre-defined criteria. 'Probably Low' was categorized as 'Low,' and 'Probably High' as 'Some concerns. Furthermore, we addressed missing evaluations; 'Other bias' domain was absent for interferon trials (RoB 1.0 tool), so we recorded it as 'Unclear' to maintain consistency. For the meta-analysis on interferon (RoB 1.0), the "Other Bias" domain was missing in the original paper, so we reviewed the paper and recorded it as 'Unclear' to ensure consistency across evaluations.

**Empirical exceptions in trial size classification**

Trial size was classified using a base-10 logarithmic comparison of sample sizes within each individual meta-analysis. In a small number of meta-analyses with highly skewed size distributions, strict application of this rule would have resulted in either excessive or minimal classification of large trials. To preserve meaningful separation, predefined empirical exceptions were applied at the meta-analysis level.

In meta-analyses with an overabundance of similarly sized trials (colchicine and convalescent plasma), trials were classified as large if their base-10 logarithm of total sample size fell within ±1 of the largest trial. Conversely, in meta-analyses with very few large trials (fluvoxamine, interferon, and anti–SARS-CoV-2 mortality), only the single largest trial was classified as large, with all others classified as small.

These exceptions were applied consistently within each meta-analysis, reviewed and agreed upon by all authors, and retained throughout subgroup and sensitivity analyses.

**Supplemental Appendix 2. PRISMA checklist^19^**

| **Section / Topic** | **Item #** | **Checklist item** | Reported location (page) |
| --- | --- | --- | --- |
| **TITLE** | | |  |
| Title | 1 | Identify the report as a systematic review. | 1 |
| **ABSTRACT** | | |  |
| Abstract | 2 | See the PRISMA 2020 for Abstracts checklist. | 5 |
| **INTRODUCTION** | | |  |
| Rationale | 3 | Describe the rationale for the review in the context of existing knowledge. | 6-7 |
| Objectives | 4 | Provide an explicit statement of the objective(s) or question(s) the review addresses. | 7 |
| **METHODS** | | |  |
| Eligibility criteria | 5 | Specify the inclusion and exclusion criteria for the review and how studies were grouped for the syntheses. | 8 |
| Information sources | 6 | Specify all databases, registers, websites, organisations, reference lists and other sources searched or consulted to identify studies. Specify the date when each source was last searched or consulted. | 8, Figure 1, eAppendix 1 |
| Search strategy | 7 | Present the full search strategies for all databases, registers and websites, including any filters and limits used. | 8, Figure 1, eAppendix 1 |
| Selection process | 8 | Specify the methods used to decide whether a study met the inclusion criteria of the review, including how many reviewers screened each record and each report retrieved, whether they worked independently, and if applicable, details of automation tools used in the process. | 3, 8-9 |
| Data collection process | 9 | Specify the methods used to collect data from reports, including how many reviewers collected data from each report, whether they worked independently, any processes for obtaining or confirming data from study investigators, and if applicable, details of automation tools used in the process. | 3, 8-9 |
| Data items | 10a | List and define all outcomes for which data were sought. Specify whether all results that were compatible with each outcome domain in each study were sought (e.g. for all measures, time points, analyses), and if not, the methods used to decide which results to collect. | 8-9 |
|  | 10b | List and define all other variables for which data were sought (e.g. participant and intervention characteristics, funding sources). Describe any assumptions made about any missing or unclear information. | 8-9, eTable 1 |
| Study risk of bias assessment | 11 | Specify the methods used to assess risk of bias in the included studies, including details of the tool(s) used, how many reviewers assessed each study and whether they worked independently, and if applicable, details of automation tools used in the process. | 3, 11, eTable 2-3 |
| Effect measures | 12 | Specify for each outcome the effect measure(s) (e.g. risk ratio, mean difference) used in the synthesis or presentation of results. | 8-11 |
| Synthesis methods | 13a | Describe the processes used to decide which studies were eligible for each synthesis (e.g. tabulating the study intervention characteristics and comparing against the planned groups for each synthesis (item #5)). | 8-11 |
|  | 13b | Describe any methods required to prepare the data for presentation or synthesis, such as handling of missing summary statistics, or data conversions. | 8-11, eAppendix 1 |
|  | 13c | Describe any methods used to tabulate or visually display results of individual studies and syntheses. | 8-11 |
|  | 13d | Describe any methods used to synthesize results and provide a rationale for the choice(s). If meta-analysis was performed, describe the model(s), method(s) to identify the presence and extent of statistical heterogeneity, and software package(s) used. | 10-11 |
|  | 13e | Describe any methods used to explore possible causes of heterogeneity among study results (e.g. subgroup analysis, meta-regression). | 9-11, eAppendix 3-4 |
|  | 13f | Describe any sensitivity analyses conducted to assess robustness of the synthesized results. | 9-11, eAppendix 3-4 |
| Reporting bias assessment | 14 | Describe any methods used to assess risk of bias due to missing results in a synthesis (arising from reporting biases). | 9-11, eTable 3 |
| Certainty assessment | 15 | Describe any methods used to assess certainty (or confidence) in the body of evidence for an outcome. | 9-11, Table 1, eTable 3 |
| **RESULTS** | | |  |
| Study selection | 16a | Describe the results of the search and selection process, from the number of records identified in the search to the number of studies included in the review, ideally using a flow diagram. | Figure 1 |
|  | 16b | Cite studies that might appear to meet the inclusion criteria, but which were excluded, and explain why they were excluded. | Appendix 1 |
| Study characteristics | 17 | Cite each included study and present its characteristics. | eTable 1, eTable 3 |
| Risk of bias in studies | 18 | Present assessments of risk of bias for each included study. | eTable 2-3 |
| Results of individual studies | 19 | For all outcomes, present, for each study: (a) summary statistics for each group (where appropriate) and (b) an effect estimate and its precision (e.g. confidence/credible interval), ideally using structured tables or plots. | eTable 1 |
| Results of syntheses | 20a | For each synthesis, briefly summarise the characteristics and risk of bias among contributing studies. | 12-15 |
|  | 20b | Present results of all statistical syntheses conducted. If meta-analysis was done, present for each the summary estimate and its precision (e.g. confidence/credible interval) and measures of statistical heterogeneity. If comparing groups, describe the direction of the effect. | 12-15, Table 1, Figure 2-4 |
|  | 20c | Present results of all investigations of possible causes of heterogeneity among study results. | eAppendix 3-4 |
|  | 20d | Present results of all sensitivity analyses conducted to assess the robustness of the synthesized results. | 12-15, eAppendix 3-4 |
| Reporting biases | 21 | Present assessments of risk of bias due to missing results (arising from reporting biases) for each synthesis assessed. | 12-15, eTable 2 |
| Certainty of evidence | 22 | Present assessments of certainty (or confidence) in the body of evidence for each outcome assessed. | 12-15, Table 1, Figure 2-4 |
| **DISCUSSION** | | |  |
| Discussion | 23a | Provide a general interpretation of the results in the context of other evidence. | 16 |
|  | 23b | Discuss any limitations of the evidence included in the review. | 18-19 |
|  | 23c | Discuss any limitations of the review processes used. | 18-10 |
|  | 23d | Discuss implications of the results for practice, policy, and future research. | 16-19 |
| **OTHER INFORMATION** | | |  |
| Registration and protocol | 24a | Provide registration information for the review, including register name and registration number, or state that the review was not registered. | 8 |
|  | 24b | Indicate where the review protocol can be accessed, or state that a protocol was not prepared. | 8 |
|  | 24c | Describe and explain any amendments to information provided at registration or in the protocol. | 8 |
| Support | 25 | Describe sources of financial or non-financial support for the review, and the role of the funders or sponsors in the review. | 3 |
| Competing interests | 26 | Declare any competing interests of review authors. | 3 |
| Availability of data, code and other materials | 27 | Report which of the following are publicly available and where they can be found: template data collection forms; data extracted from included studies; data used for all analyses; analytic code; any other materials used in the review. | 3 |

**Supplemental Appendix 3. Results of heterogeneity investigation**

For the ROR meta-analysis, the original heterogeneity results indicated moderate variability across studies (I² = 55.4%, τ² = 0.034, Q test P-value < 0.001). A subgroup analysis was then performed, with 19 meta-analyses that included more than 2 trials. This subgroup analysis revealed no variability across studies (I² = 0%, τ² = 0.002, Q test P-value = 0.487). The pooled ROR was 0.89 (95% CI: 0.83 to 0.96) with a significant P-value of 0.001.

With the 1,000-participant threshold, the original heterogeneity was moderate (I² = 57.5%, τ² = 0.066; Q-test P = 0.223 for Egger shows no small-study effects). After restricting to meta-analyses with >2 trials (excluding 1-large/1-small pairs), heterogeneity was markedly reduced (I² = 9.0%, τ² = 0.001; Q-test P = 0.345), with a significant pooled effect (ROR = 0.91, 95% CI 0.85–0.97; P = 0.004).

With the median-based threshold, the original heterogeneity was also moderate (I² = 50.9%, τ² = 0.103; Q-test; Egger P = 0.553). After restricting to meta-analyses with >2 trials, heterogeneity disappeared (I² = 0%, τ² = 0.002; Q-test P = 0.732), and the pooled effect remained directionally consistent (ROR = 0.83, 95% CI 0.74–0.93; P = 0.001).

Six meta-analyses were excluded from the subgroup analysis due to containing only one large and one small trial: two evaluating anti-SARS-CoV-2 monoclonal antibodies for mortality (in seronegative and seropositive patients), two assessing monoclonal antibodies for symptom progression (in seronegative and overall populations), one on azithromycin for mortality in severe cases, and one on canakinumab for mortality.

We believe that the heterogeneity observed in the initial analysis was driven by results from the 6 meta-analyses with only one small and one large trial. DerSimonian-Laird random-effects models were used to account for these variabilites.

**Supplemental Appendix 4. Sensitivity and subgroup analyses using alternative definitions of trial size and random-effects estimators**

**1. Sensitivity Analysis by Alternative Sample Size Definitions (ROR)**

**Fixed cutoff of 1000 participants:**
Using a sample size threshold of 1000 participants to distinguish large and small trials, the pooled ratio of odds ratios (ROR) was 0.84 (95% CI: 0.73–0.98; P = 0.023), indicating that small trials yielded more favorable results. Heterogeneity was moderate (I² = 57.5%; τ² = 0.066), and the Egger test showed no evidence of small-study effects (P = 0.223). The overall effect test was statistically significant (Z = -2.28). Kappa statistics indicated weak agreement between small and large trials (κ = -0.220, P = 0.166) and between small and all trials (κ = -0.176, P = 0.356), while agreement between large and all trials was substantial (κ = 0.615, P = 0.001).

**Median-based cutoff:**
When using the median sample size to dichotomize trials, the ROR was 0.78 (95% CI: 0.64–0.94; P = 0.008), similarly demonstrating greater effects among small trials. Heterogeneity remained moderate (I² = 50.9%; τ² = 0.103), and no significant small-study effect was detected (Egger P = 0.553). The overall effect test yielded Z = -2.64. Kappa agreement remained low between small and large trials (κ = 0.123, P = 0.539) and between small and all trials (κ = 0.186, P = 0.349), while large vs. all trials showed strong agreement (κ = 0.884, P < 0.001).

**2. Subgroup Analysis by Outcome Type**

**Fixed cutoff of 1000 participants:**
For trials assessing all-cause mortality, the pooled ROR was 0.91 (95% CI: 0.85–0.98; P = 0.013), showing significantly greater treatment effects in small trials. For non-mortality outcomes, the pooled ROR was 0.51 (95% CI: 0.19–1.36; P = 0.175). While not statistically significant for non-mortality outcomes, the trend toward stronger effects in small trials persisted.

**Median-based cutoff:**
The ROR for all-cause mortality was 0.85 (95% CI: 0.74–0.96; P = 0.010), whereas for non-mortality outcomes, it was 0.42 (95% CI: 0.17–1.07; P = 0.070). While not statistically significant for non-mortality outcomes, the trend toward stronger effects in small trials persisted.

**3. Subgroup Analysis by Treatment Type**

**Fixed cutoff of 1000 participants:**
The pooled RORs were 0.97 (95% CI: 0.84–1.12; P = 0.673) for anti-infective agents, 0.97 (95% CI: 0.89–1.07; P = 0.605) for anti-inflammatory and immunomodulatory agents, 0.49 (95% CI: 0.25–0.96; P = 0.037) for anti-SARS-CoV-2 monoclonal antibodies, and 0.79 (95% CI: 0.62–1.03; P = 0.078) for miscellaneous agents.

**Median-based cutoff:**
The respective pooled RORs were 1.07 (95% CI: 0.88–1.30; P = 0.470) for anti-infective agents, 0.88 (95% CI: 0.73–1.06; P = 0.185) for anti-inflammatory and immunomodulatory agents, 0.48 (95% CI: 0.25–0.94; P = 0.032) for anti-SARS-CoV-2 monoclonal antibodies, and 0.67 (95% CI: 0.54–0.84; P < 0.001) for miscellaneous agents.

**4. Time-Restricted ROR Analysis (Small Trials Published Prior to Large Trials)**

**Fixed cutoff of 1000 participants:**
After excluding one meta-analysis ("IL-1 receptor blockers—mortality") in which all small trials were published after the first large trial, the pooled ROR for small trials published prior to the first large trial was 0.82 (95% CI: 0.69–0.96; P = 0.012), indicating significantly more favorable effects in earlier small trials. Heterogeneity was moderate (I² = 58.2%; τ² = 0.077), and the overall effect was statistically significant (Z = –2.51).

**Median-based cutoff:**
When using the median sample size for classification, the pooled ROR among earlier small trials was 0.68 (95% CI: 0.51–0.90; P = 0.007), with moderate heterogeneity (I² = 57.8%; τ² = 0.213). The overall effect was again significant (Z = -2.68).

**5. Sensitivity Analysis Using Alternative Random-Effects Models**

To assess the robustness of the findings to the choice of random-effects estimator and variance correction, we conducted sensitivity analyses using multiple established approaches. Using the restricted maximum likelihood estimator with Hartung–Knapp adjustment, the pooled ROR was 0.86 (95% CI, 0.75–0.98; P = 0.027). A similar estimate was obtained with the DerSimonian–Laird estimator with Hartung–Knapp adjustment (ROR, 0.86; 95% CI, 0.76–0.98; P = 0.028). Analyses based on the Paule–Mandel estimator with Hartung–Knapp adjustment (ROR, 0.82; 95% CI, 0.70–0.98; P = 0.029) and the Sidik–Jonkman estimator with Hartung–Knapp adjustment (ROR, 0.81; 95% CI, 0.67–0.97; P = 0.026) yielded consistent results.

When the Hartung–Knapp correction was not applied, the direction and statistical significance of the effect remained unchanged. The restricted maximum likelihood estimator without Hartung–Knapp adjustment produced an ROR of 0.85 (95% CI, 0.76–0.95; P = 0.003), while the DerSimonian–Laird estimator without Hartung–Knapp adjustment yielded an identical estimate (ROR, 0.85; 95% CI, 0.76–0.95; P = 0.004). Results were also consistent using the Paule–Mandel estimator without Hartung–Knapp adjustment (ROR, 0.82; 95% CI, 0.69–0.97; P = 0.018) and the Sidik–Jonkman estimator without Hartung–Knapp adjustment (ROR, 0.80; 95% CI, 0.66–0.98; P = 0.029).

**6. Fragility Metrics and Risk of Bias Profiles**

**Fixed cutoff of 1000 participants:**
Using the ≥1,000-participant threshold, large trials showed greater statistical stability, with higher median FI (14.0 vs. 4.0; *P* = 0.018) and RFI (18.0 vs. 5.0; *P* < 0.001), and generally lower risk of bias, though differences across RoB 2.0 domains were not statistically significant.

**Median-based cutoff:**
When applying a median-based cutoff, large trials again demonstrated greater robustness (FI: 11.0 vs. 3.5; *P* = 0.028; RFI: 12.6 vs. 4.0; *P* < 0.001) and significantly lower risk of bias in Domains 1 (randomization), 3 (missing outcome data), and in overall assessments. These results are shown in **Supplementary Figure 2**.

**Supplemental Figure 1. Forest plot of pooled meta-analysis by study size**


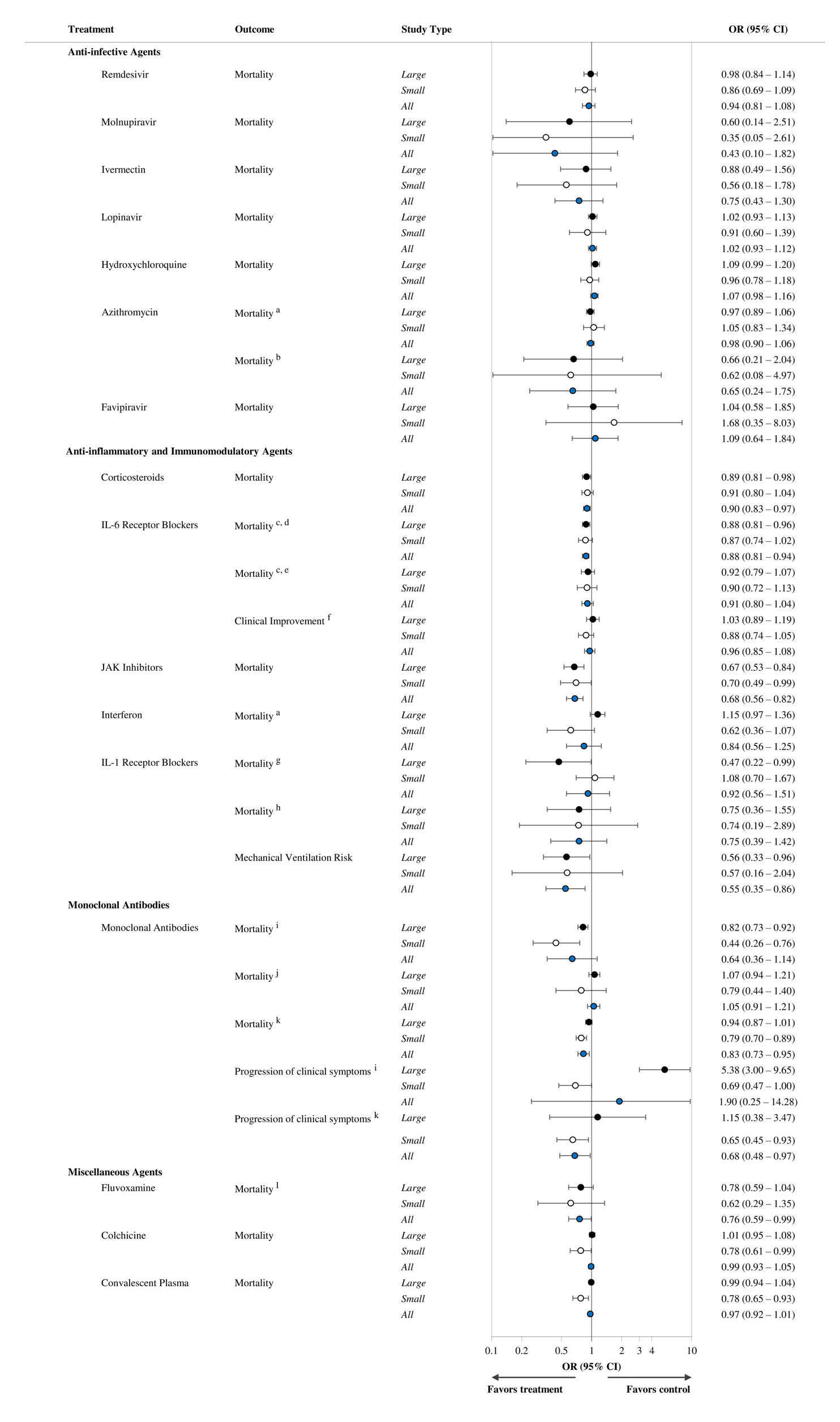


The black dots show the pooled OR for large trials, the white dots for small trials, and the blue dots for all. The arrows indicate when the effect size interval exceeds the range of 0.1 to 10.

**Abbreviations:** OR, Odds Ratio; CI, Confidence Interval; IL, Interleukin.

**Footnotes:** ^a^ Severe patients, ^b^ Non-severe patients, ^c^ Tocilizumab, ^d^ All-cause mortality at day 28, ^e^ All-cause mortality at day 60, ^f^ Sarilumab, ^g^ Anakinra, ^h^ Canakinumab, ^i^ Seronegative baseline patients, ^j^ Seropositive baseline patients, ^k^ Overall baseline patients, ^l^ Inpatients.

**Supplemental Figure 2. Scatter plot of effect size comparisons between pre-large and post-large trials**





Each point represents a meta-analysis, comparing the effect size from post-large trials (x-axis) and pre-large trials (y-axis). Points are colored by treatment group: anti-infective agents (blue), anti-inflammatory and immunomodulatory agents (red), anti-SARS-CoV-2 monoclonal antibodies (green), and miscellaneous agents (cyan). The dotted diagonal line represents equal effect estimates from large and small trials. Points above the line indicate a greater benefit in post-large trials, while points below the line indicate a greater benefit in pre-large trials. The marginal density plots illustrate the distribution of effect sizes by trial size and treatment group.

**Abbreviations:** Exp, Exponential.

**Supplemental Figure 3**. **Stability and bias profiles by trial size across alternative thresholds**

**(A)** **Distributions of FI and RFI in large and small trials (≥1,000-Participant Threshold)**


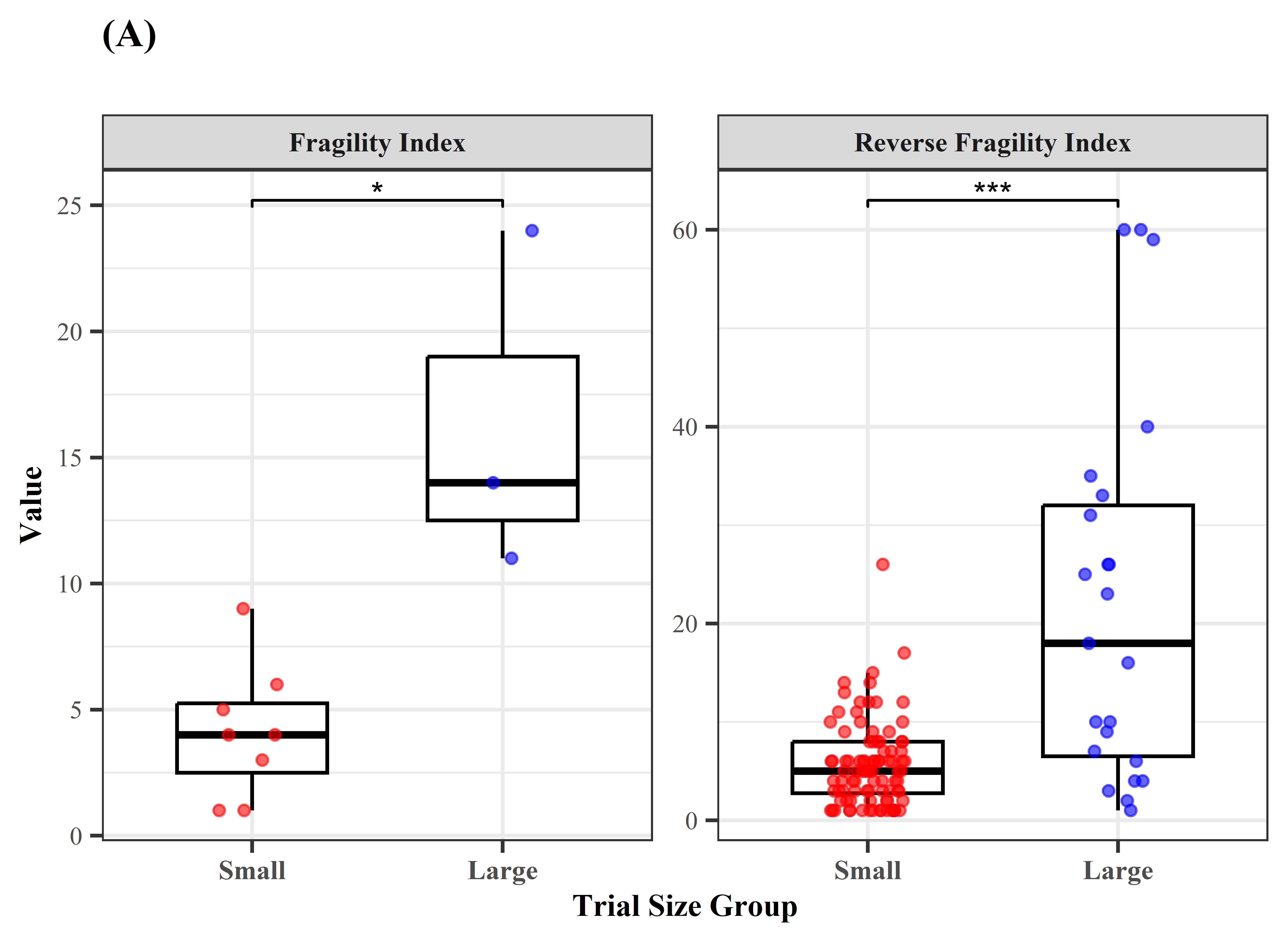


**(B)** **Distributions of FI and RFI in large and small trials (Median-Based Threshold)**


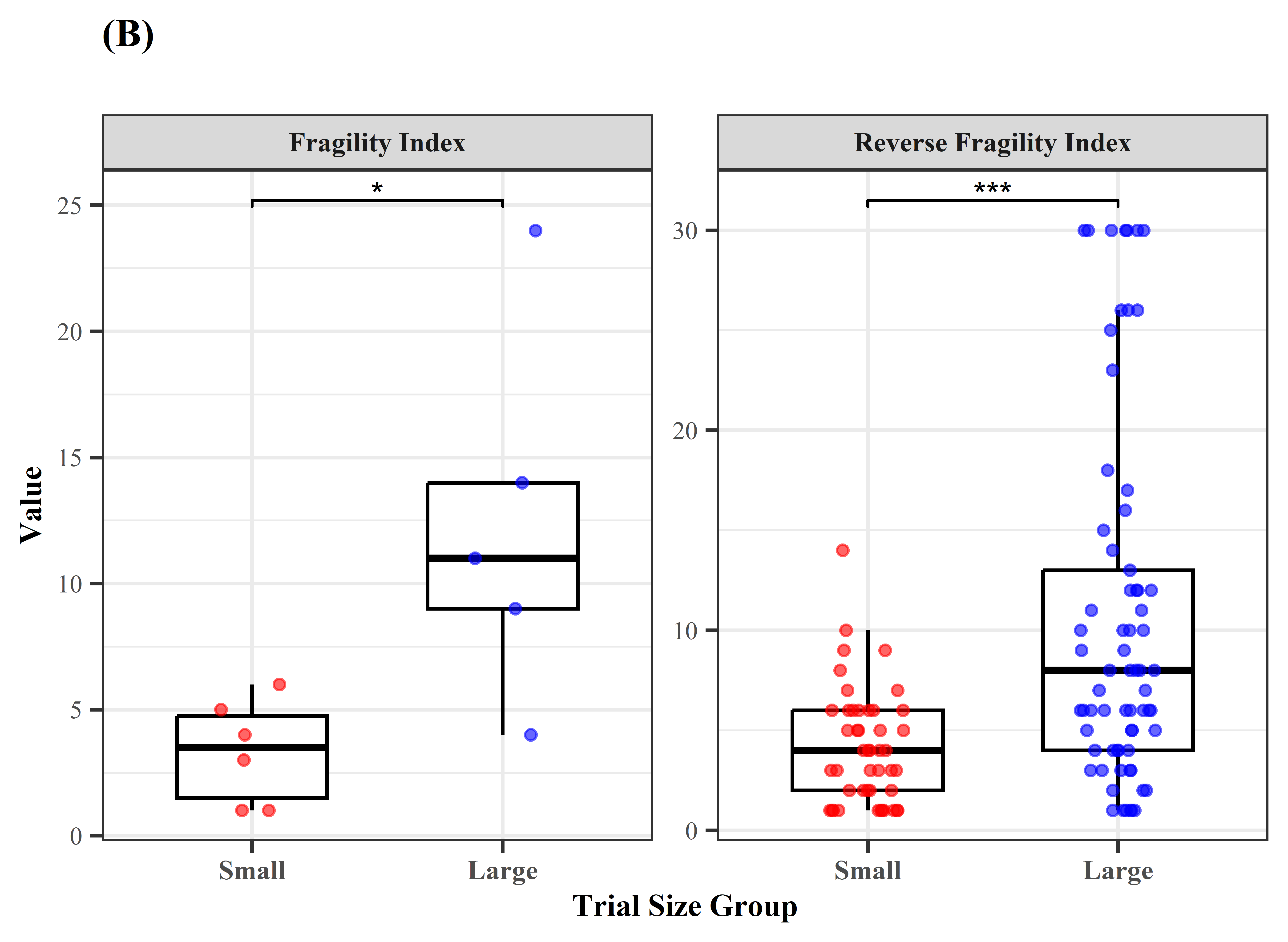


**(C)** **Distribution of RoB 2.0 Assessments in large and small trials (≥1,000-Participant Threshold)**


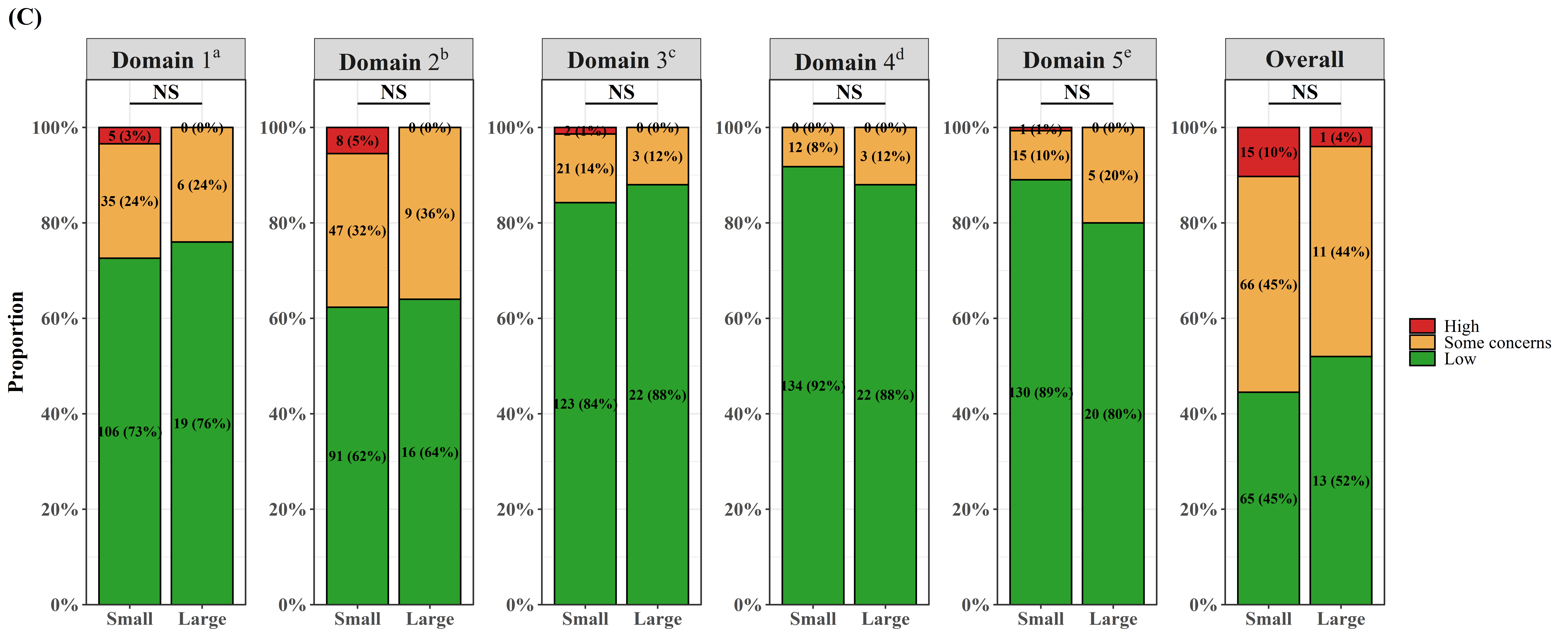


**(D)** **Distribution of RoB 2.0 Assessments in large and small trials** **(Median-Based Threshold)**


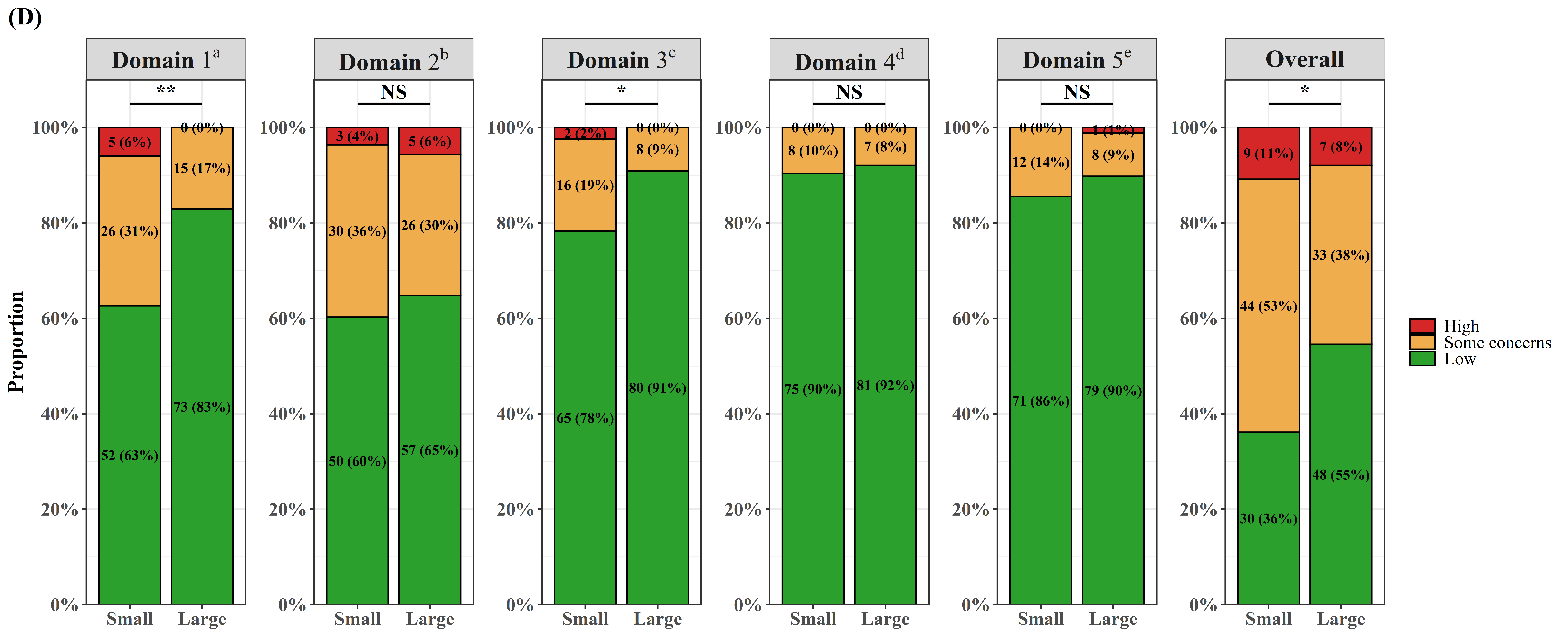


**Abbreviations:** FI, Fragility Index; NS, Not Significant; RFI, Reverse Fragility Index

**Footnotes:** ^a^ Bias arising from the randomization process, ^b^ Bias due to deviations from intended intervention, ^c^ Bias due to missing outcome data, ^d^ Bias in measurement of the outcome, ^e^ Bias in selection of the reported result.

**Supplemental Table 1. Individual study statistics**

**(A) Individual study statistics of anti-infective agents**

| **Treatment** | **Outcome** | **Study Name** | **Study Year** | **Treatment**  **(Event / Population)** | **Control**  **(Event / Population)** | **Total**  **(Event/**  **Population)** | ***P*** | **FI** | **RFI** | **Large** |
| --- | --- | --- | --- | --- | --- | --- | --- | --- | --- | --- |
| Remdesivir^1^ | Mortality | Total | | 433/4175 | 442/4060 | 875/8235 | NA | | | |
|  |  | Christoph D. Spinner et al | 2020 | 3/193 | 4/200 | 7/393 | 1.000 | 0 | 3 | 0 |
|  |  | Florence Ader et al | 2022 | 34/414 | 38/418 | 72/832 | 0.712 | 0 | 12 | 0 |
|  |  | J.H. Beigel et al | 2020 | 59/541 | 77/521 | 136/1062 | 0.066 | 0 | 2 | 0 |
|  |  | Lakshmi Mahajan et al | 2021 | 5/34 | 3/36 | 8/70 | 0.472 | 0 | 3 | 0 |
|  |  | Sherief Abd-Elsalam et al | 2022 | 9/100 | 7/100 | 16/200 | 0.795 | 0 | 6 | 0 |
|  |  | WHO Solidarity Trial Consortium | 2021 | 301/2743 | 303/2708 | 604/5451 | 0.829 | 0 | 40 | 1 |
|  |  | Yeming Wang et al | 2020 | 22/150 | 10/77 | 32/227 | 0.841 | 0 | 6 | 0 |
| Molnupiravir^2^ | Mortality | Total | | 4/14987 | 16/14701 | 20/29688 | NA | | | |
|  |  | Arribas et al | 2021 | 13/218 | 1/75 | 14/293 | 0.127 | 0 | 1 | 0 |
|  |  | Butler et al | 2022 | 3/12529 | 5/12525 | 8/25054 | 0.508 | 0 | 3 | 1 |
|  |  | Caraco et al | 2022 | 0/225 | 1/74 | 1/299 | 0.247 | 0 | 0 | 0 |
|  |  | Fischer II et al | 2022 | 0/140 | 1/62 | 1/202 | 0.307 | 0 | 0 | 0 |
|  |  | Bernal et al | 2022 | 1/709 | 9/699 | 10/1408 | 0.011 | 2 | 0 | 0 |
|  |  | Khoo et al | 2022 | 0/90 | 0/90 | 0/180 | 1.000 | 0 | 0 | 0 |
|  |  | Sinha et al | 2022 | 0/608 | 0/610 | 0/1218 | 1.000 | 0 | 0 | 0 |
|  |  | Tippabhotla et al | 2022 | 0/610 | 0/610 | 0/1220 | 1.000 | 0 | 0 | 0 |
|  |  | Zou et al | 2022 | 0/76 | 0/31 | 0/107 | 1.000 | 0 | 0 | 0 |
| Ivermectin^3^ | Mortality | Total | | 28/1440 | 38/1420 | 66/2860 | NA | | | |
|  |  | Bounfrate et al | 2021 | 0/58 | 0/31 | 0/89 | 1.000 | 0 | 0 | 0 |
|  |  | Chaccour et al | 2021 | 0/12 | 0/12 | 0/24 | 1.000 | 0 | 0 | 0 |
|  |  | I-TECH | 2022 | 3/241 | 10/249 | 13/490 | 0.089 | 0 | 1 | 0 |
|  |  | Lopez-Medina et al | 2021 | 0/200 | 1/198 | 1/398 | 0.497 | 0 | 0 | 0 |
|  |  | TOGETHER | 2022 | 21/679 | 24/679 | 45/1358 | 0.762 | 0 | 10 | 1 |
|  |  | Vallejos et al | 2021 | 4/250 | 3/251 | 7/501 | 0.724 | 0 | 3 | 0 |
| Lopinavir^4^ | Mortality | Total | | 557/3549 | 951/5296 | 1508/8845 | NA | | | |
|  |  | Ader et al | 2021 | 14/145 | 12/148 | 26/293 | 0.685 | 0 | 7 | 0 |
|  |  | Cao et al | 2020 | 19/99 | 25/100 | 44/199 | 0.394 | 0 | 6 | 0 |
|  |  | Horby et al | 2020 | 374/1616 | 767/3424 | 1141/5040 | 0.564 | 0 | 59 | 1 |
|  |  | Li et al | 2020 | 0/34 | 0/17 | 0/51 | 1.000 | 0 | 0 | 0 |
|  |  | Pan et al | 2020 | 148/1411 | 146/1380 | 294/2791 | 0.951 | 0 | 31 | 1 |
|  |  | Reis et al | 2020 | 2/244 | 1/227 | 3/471 | 1.000 | 0 | 1 | 0 |
| Hydroxy-chloroquine^4^ | Mortality | Total | | 656/4487 | 1016/6070 | 1672/10557 | NA | | | |
|  |  | Abd-Elsalam et al | 2020 | 6/97 | 5/97 | 11/194 | 1.000 | 0 | 5 | 0 |
|  |  | Ader et al | 2021 | 11/145 | 12/148 | 23/293 | 1.000 | 0 | 8 | 0 |
|  |  | REMAP-CAP | 2020 | 17/61 | 22/81 | 39/142 | 1.000 | 0 | 12 | 0 |
|  |  | HYDRA | 2020 | 0/5 | 0/3 | 0/8 | 1.000 | 0 | 0 | 0 |
|  |  | CCAP-1 | 2020 | 1/4 | 0/2 | 1/6 | 1.000 | 0 | 0 | 0 |
|  |  | COMHY | 2020 | 0/8 | 0/8 | 0/16 | 1.000 | 0 | 0 | 0 |
|  |  | COV-HCQ | 2020 | 1/13 | 0/14 | 1/27 | 0.481 | 0 | 0 | 0 |
|  |  | CCAP-1 2 | 2020 | 0/1 | 0/1 | 0/2 | 1.000 | 0 | 0 | 0 |
|  |  | OAHU-COVID19 | 2020 | 2/10 | 0/6 | 2/16 | 0.500 | 0 | 0 | 0 |
|  |  | ARCHAIC | 2020 | 2/4 | 0/6 | 2/10 | 0.133 | 0 | 0 | 0 |
|  |  | Cavalcanti et al | 2020 | 9/221 | 8/227 | 17/448 | 0.809 | 0 | 6 | 0 |
|  |  | Chen et al (3) | 2020 | 0/15 | 0/15 | 0/30 | 1.000 | 0 | 0 | 0 |
|  |  | Chen et al (4) | 2020 | 0/18 | 0/12 | 0/30 | 1.000 | 0 | 0 | 0 |
|  |  | Chen et al (5) | 2020 | 0/21 | 0/12 | 0/33 | 1.000 | 0 | 0 | 0 |
|  |  | Dubee et al | 2021 | 6/124 | 11/123 | 17/247 | 0.220 | 0 | 3 | 0 |
|  |  | Gonzalez et al | 2021 | 2/33 | 6/37 | 8/70 | 0.266 | 0 | 2 | 0 |
|  |  | Hernadex-Cardenas et al | 2021 | 40/106 | 44/108 | 84/214 | 0.676 | 0 | 11 | 0 |
|  |  | Horby et al (2) | 2020 | 421/1561 | 790/3155 | 1211/4716 | 0.157 | 0 | 23 | 1 |
|  |  | Johnston et al | 2021 | 0/71 | 0/83 | 0/154 | 1.000 | 0 | 0 | 0 |
|  |  | Lyngbakken et al | 2020 | 1/26 | 1/25 | 2/51 | 1.000 | 0 | 1 | 0 |
|  |  | Omrani et al | 2020 | 0/152 | 0/152 | 0/304 | 1.000 | 0 | 0 | 0 |
|  |  | Pan et al | 2020 | 104/947 | 84/906 | 188/1853 | 0.248 | 0 | 9 | 1 |
|  |  | PATCH | 2020 | 0/15 | 0/15 | 0/30 | 1.000 | 0 | 0 | 0 |
|  |  | TOGETHER | 2020 | 0/214 | 1/227 | 1/441 | 1.000 | 0 | 0 | 0 |
|  |  | Self et al | 2020 | 25/242 | 25/237 | 50/479 | 1.000 | 0 | 12 | 0 |
|  |  | Skipper et al | 2020 | 1/231 | 1/234 | 2/465 | 1.000 | 0 | 1 | 0 |
|  |  | Tang et al | 2020 | 0/75 | 0/75 | 0/150 | 1.000 | 0 | 0 | 0 |
|  |  | Ulrich et al | 2020 | 7/67 | 6/61 | 13/128 | 1.000 | 0 | 6 | 0 |
| Azithromycin^5^ | Mortality^a^ | Total | | 651/2796 | 1235/5364 | 1686/8160 | NA | | | |
|  |  | Furtado et al | 2020 | 90/214 | 73/183 | 163/397 | 0.683 | 0 | 14 | 0 |
|  |  | Horby et al | 2021 | 561/2582 | 1162/5181 | 1723/7763 | 0.505 | 0 | 33 | 1 |
|  | Mortality^b^ | Total | | 6/304 | 9/290 | 15/594 | NA | | | |
|  |  | Cavalcanti et al | 2020 | 5/172 | 7/159 | 12/331 | 0.562 | 0 | 4 | 1 |
|  |  | Sekhavati et al | 2020 | 0/56 | 1/55 | 1/111 | 0.495 | 0 | 0 | 0 |
|  |  | Hinks et al | 2021 | 1/76 | 1/76 | 2/152 | 1.000 | 0 | 1 | 0 |
| Favipiravir^6^ | Mortality | Total | | 49/1081 | 46/993 | 95/2074 | NA | | | |
|  |  | AlQahtani et al | 2022 | 1/54 | 0/52 | 1/106 | 1.000 | 0 | 0 | 0 |
|  |  | Chuah et al | 2022 | 5/250 | 0/250 | 5/500 | 0.061 | 0 | 0 | 1 |
|  |  | Finberg et al | 2021 | 1/25 | 0/25 | 1/50 | 1.000 | 0 | 0 | 0 |
|  |  | Ivashchenko et al | 2021 | 2/40 | 0/20 | 2/60 | 0.548 | 0 | 0 | 0 |
|  |  | Pushkar et al | 2020 | 0/100 | 0/100 | 0/200 | 1.000 | 0 | 0 | 1 |
|  |  | Shah et al | 2023 | 26/221 | 34/225 | 60/446 | 0.333 | 0 | 7 | 1 |
|  |  | Shenoy et al | 2021 | 14/175 | 11/178 | 25/353 | 0.540 | 0 | 6 | 1 |
|  |  | Shinkai et al | 2021 | 0/107 | 0/49 | 0/156 | 1.000 | 0 | 0 | 1 |
|  |  | Udwadia et al | 2021 | 0/73 | 1/75 | 1/148 | 1.000 | 0 | 0 | 0 |
|  |  | Zhao et al | 2021 | 0/36 | 0/19 | 0/55 | 1.000 | 0 | 0 | 0 |

**(B) Individual study statistics of anti-inflammatory and immunomodulatory agents**

| **Treatment** | **Outcome** | **Study Name** | **Study Year** | **Treatment**  **(Event / Population)** | **Control**  **(Event / Population)** | **Total (Event/**  **Population)** | ***P*** | **FI** | **RFI** | **Large** |
| --- | --- | --- | --- | --- | --- | --- | --- | --- | --- | --- |
| Cortico-  steroids^7^ | Mortality | Total | | 732/4020 | 1382/6461 | 2114/10481 | NA | | | |
|  |  | Angus et al | 2020 | 41/137 | 33/101 | 74/238 | 0.673 | 0 | 13 | 0 |
|  |  | Clemency et al | 2022 | 0/197 | 0/203 | 0/400 | 1.000 | 0 | 0 | 0 |
|  |  | Dequin et al | 2020 | 11/76 | 20/73 | 31/149 | 0.069 | 0 | 1 | 0 |
|  |  | Edalatifard et al | 2020 | 2/34 | 12/28 | 14/62 | 0.001 | 5 | 0 | 0 |
|  |  | Ezer et al | 2021 | 0/105 | 0/98 | 0/203 | 1.000 | 0 | 0 | 0 |
|  |  | Ghanel et al | 2021 | 4/116 | 6/110 | 10/226 | 0.530 | 0 | 4 | 0 |
|  |  | Jamaati et al | 2021 | 16/25 | 15/25 | 31/50 | 1.000 | 0 | 7 | 0 |
|  |  | Jeronimo et al | 2021 | 72/194 | 76/199 | 148/393 | 0.836 | 0 | 17 | 0 |
|  |  | Luis Corral-Gudino et al | 2021 | 7/35 | 5/29 | 12/64 | 1.000 | 0 | 5 | 0 |
|  |  | Marie et al | 2021 | 6/16 | 2/14 | 8/30 | 0.226 | 0 | 2 | 0 |
|  |  | RECOVERY Collaborative Group | 2021 | 482/2104 | 1110/4321 | 1592/6425 | 0.016 | 11 | 0 | 1 |
|  |  | Tang et al | 2021 | 0/43 | 1/43 | 1/86 | 1.000 | 0 | 0 | 0 |
|  |  | Tomazini et al | 2020 | 85/151 | 91/148 | 176/299 | 0.411 | 0 | 10 | 0 |
|  |  | Yu et al | 2021 | 6/787 | 11/1069 | 17/1856 | 0.629 | 0 | 5 | 0 |
| IL-6 Receptor Blockers^8^ | Mortality^c,d^ | Total | | 905/3880 | 1012/3574 | 1917/7454 | NA | | | |
|  |  | Declercq J et al | 2021 | 9/82 | 7/72 | 16/154 | 1.000 | 0 | 6 | 0 |
|  |  | IMMCOVA | 2021 | 2/22 | 2/27 | 4/49 | 1.000 | 0 | 2 | 0 |
|  |  | HMO-0224-20 | 2021 | 11/37 | 8/17 | 19/54 | 0.237 | 0 | 4 | 0 |
|  |  | COVITOZ-01 | 2021 | 0/17 | 0/9 | 0/26 | 1.000 | 0 | 0 | 0 |
|  |  | Broman N et al | 2022 | 1/59 | 0/29 | 1/88 | 1.000 | 0 | 0 | 0 |
|  |  | COVIDOSE-2 | 2021 | 0/20 | 2/8 | 2/28 | 0.074 | 0 | 0 | 0 |
|  |  | Hermine O et al | 2022 | 8/51 | 10/46 | 18/97 | 0.602 | 0 | 5 | 0 |
|  |  | ARCHITECTS | 2021 | 0/10 | 2/11 | 2/21 | 0.476 | 0 | 0 | 0 |
|  |  | Talaschian M et al | 2021 | 5/20 | 4/20 | 9/40 | 1.000 | 0 | 4 | 0 |
|  |  | Rutgers A et al | 2021 | 21/174 | 34/180 | 55/354 | 0.080 | 0 | 2 | 0 |
|  |  | Soin AS et al | 2021 | 13/90 | 15/90 | 28/180 | 0.837 | 0 | 8 | 0 |
|  |  | Horby P et al | 2021 | 621/2022 | 729/2094 | 1350/4116 | 0.005 | 24 | 0 | 1 |
|  |  | Veiga VC et al | 2021 | 14/65 | 6/64 | 20/129 | 0.087 | 0 | 1 | 0 |
|  |  | Gordon AC et al | 2021 | 98/366 | 142/412 | 240/778 | 0.024 | 4 | 0 | 0 |
|  |  | Salama C et al | 2020 | 26/259 | 11/129 | 37/388 | 0.716 | 0 | 6 | 0 |
|  |  | Stone JH et al | 2020 | 9/161 | 3/82 | 12/243 | 0.756 | 0 | 3 | 0 |
|  |  | Salvarani C et al | 2020 | 2/60 | 1/66 | 3/126 | 0.605 | 0 | 1 | 0 |
|  |  | Hermine O et al | 2020 | 7/64 | 8/67 | 15/131 | 1.000 | 0 | 6 | 0 |
|  |  | Rosas IO et al | 2022 | 58/301 | 28/151 | 86/452 | 0.899 | 0 | 11 | 0 |
|  | Mortality^c,e^ | Total | | 472/1852 | 248/949 | 720/2801 | NA | | | |
|  |  | Declercq J et al | 2021 | 10/82 | 9/72 | 19/154 | 1.000 | 0 | 8 | 0 |
|  |  | HMO-0224-20 | 2021 | 18/37 | 10/17 | 28/54 | 0.565 | 0 | 8 | 0 |
|  |  | COVITOZ-01 | 2021 | 0/17 | 0/9 | 0/26 | 1.000 | 0 | 0 | 0 |
|  |  | Broman N et al | 2022 | 1/59 | 1/29 | 2/88 | 1.000 | 0 | 1 | 0 |
|  |  | Hermine O et al | 2022 | 12/51 | 13/46 | 25/97 | 0.647 | 0 | 7 | 0 |
|  |  | ARCHITECTS | 2021 | 0/10 | 2/11 | 2/21 | 0.476 | 0 | 0 | 0 |
|  |  | Derde L et al | 2021 | 323/972 | 151/418 | 474/1390 | 0.324 | 0 | 26 | 1 |
|  |  | Salama C et al | 2020 | 29/259 | 15/129 | 44/388 | 1.000 | 0 | 15 | 0 |
|  |  | Hermine O et al | 2020 | 7/64 | 11/67 | 18/131 | 0.450 | 0 | 4 | 0 |
|  |  | Rosas IO et al | 2022 | 72/301 | 36/151 | 108/452 | 1.000 | 0 | 13 | 0 |
|  | Clinical improve-ment^f^ | Total | | 702/1747 | 242/678 | 944/2425 | NA | | | |
|  |  | Branch-Elliman W et al | 2022 | 5/20 | 3/30 | 8/50 | 0.240 | 0 | 2 | 0 |
|  |  | Merchante N et al | 2021 | 13/79 | 7/39 | 20/118 | 1.000 | 0 | 9 | 0 |
|  |  | Garcia-Vicuna R et al | 2022 | 3/20 | 0/10 | 3/30 | 0.532 | 0 | 0 | 0 |
|  |  | Sancho-Lopez A et al | 2021 | 9/99 | 8/102 | 17/201 | 0.804 | 0 | 6 | 0 |
|  |  | Hermine O et al | 2022 | 29/50 | 29/41 | 58/91 | 0.274 | 0 | 5 | 0 |
|  |  | Mariette X et al | 2021 | 17/68 | 27/80 | 44/148 | 0.282 | 0 | 5 | 0 |
|  |  | Sivapalasingam et al | 2022 | 473/1044 | 126/286 | 599/1330 | 0.738 | 0 | 16 | 1 |
|  |  | Sivapalasingam et al 2 | 2022 | 153/367 | 42/90 | 195/457 | 0.407 | 0 | 26 | 0 |
| JAK Inhibitors^9^ | Mortality | Total | | 147/1985 | 214/1959 | 361/3944 | NA | | | |
|  |  | Bari/EU-SolidAct | 2022 | 14/139 | 18/136 | 32/275 | 0.456 | 0 | 6 | 0 |
|  |  | Kalil et al | 2021 | 24/515 | 37/518 | 61/1033 | 0.113 | 0 | 3 | 1 |
|  |  | Marconi et al | 2021 | 62/764 | 100/761 | 162/1525 | 0.002 | 14 | 0 | 1 |
|  |  | Wesley-Ely et al | 2021 | 20/51 | 29/50 | 49/101 | 0.074 | 0 | 1 | 0 |
|  |  | Wolfe et al | 2022 | 27/516 | 30/494 | 57/1010 | 0.588 | 0 | 10 | 1 |
| Interferon^10^ | Mortality^a^ | Total | | 285/2160 | 261/2137 | 546/4297 | NA | | | |
|  |  | Ader et al | 2021 | 13/54 | 10/51 | 23/105 | 0.642 | 0 | 6 | 0 |
|  |  | Alavi Darazam et al | 2021 | 10/40 | 9/20 | 19/60 | 0.146 | 0 | 3 | 0 |
|  |  | Davoudi-Monfared et al | 2020 | 8/42 | 17/39 | 25/81 | 0.029 | 1 | 0 | 0 |
|  |  | Kalil et al | 2021 | 19/403 | 16/414 | 35/817 | 0.606 | 0 | 8 | 1 |
|  |  | Pan et al | 2021 | 233/1568 | 203/1560 | 436/3128 | 0.148 | 0 | 10 | 1 |
|  |  | Pandit et al | 2021 | 0/20 | 0/20 | 0/40 | 1.000 | 0 | 0 | 0 |
|  |  | Rahmani et al | 2020 | 2/33 | 6/33 | 8/66 | 0.258 | 0 | 2 | 0 |
| IL-1 Receptor Blockers^11^ | Mortality^g^ | Total | | 47/560 | 45/367 | 92/927 | NA | | | |
|  |  | Audemard-Verger et al | 2022 | 9/37 | 3/34 | 12/71 | 0.115 | 0 | 1 | 0 |
|  |  | CORIMUNO-19 Collaborative group | 2021 | 13/59 | 13/55 | 26/114 | 1.000 | 0 | 8 | 0 |
|  |  | Declercq et al | 2021 | 7/44 | 9/74 | 16/118 | 0.587 | 0 | 6 | 0 |
|  |  | Kharazmi et al | 2021 | 5/15 | 7/15 | 12/30 | 0.710 | 0 | 4 | 0 |
|  |  | Kyriazopoulou et al | 2021 | 13/405 | 13/189 | 26/594 | 0.052 | 0 | 1 | 1 |
|  | Mortality^h^ | Total | | 16/256 | 19/243 | 35/499 | NA | | | |
|  |  | Caricchio et al | 2021 | 12/227 | 16/227 | 28/454 | 0.559 | 0 | 6 | 1 |
|  |  | NCT04365153 | 2022 | 4/29 | 3/16 | 7/45 | 0.686 | 0 | 4 | 0 |
|  | Risk of requiring mechanical ventilation | Total | | 31/741 | 43/515 | 74/1256 | NA | | | |
|  |  | Audemard-Verger et al | 2022 | 0/35 | 1/32 | 1/67 | 0.478 | 0 | 0 | 0 |
|  |  | Caricchio et al | 2021 | 11/227 | 18/224 | 29/451 | 0.183 | 0 | 3 | 1 |
|  |  | CORIMUNO-19 Collaborative group | 2021 | 5/59 | 3/55 | 8/114 | 0.718 | 0 | 3 | 0 |
|  |  | Kharazmi et al | 2021 | 3/15 | 10/15 | 13/30 | 0.025 | 1 | 0 | 0 |
|  |  | Kyriazopoulou et al | 2021 | 12/405 | 11/189 | 23/594 | 0.110 | 0 | 3 | 1 |

**(C) Individual study statistics of anti-SARS-CoV-2 monoclonal antibodies**

| **Treatment** | **Outcome** | **Study Name** | **Study Year** | **Treatment**  **(Event / Population)** | **Control**  **(Event / Population)** | **Total (Event/**  **Population)** | ***P*** | **FI** | **RFI** | **Large** |
| --- | --- | --- | --- | --- | --- | --- | --- | --- | --- | --- |
| Anti-SARS-CoV-2 monoclonal antibodies^12^ | Mortality^i^ | Total | | 420/1993 | 476/1680 | 896/3673 | NA | | | |
|  |  | Horby and Landray et al | 2022 | 396/1633 | 452/1520 | 848/3153 | 0.001 | 38 | 0 | 1 |
|  |  | Somersan-Karakaya et al | 2022 | 24/360 | 24/160 | 48/520 | 0.005 | 9 | 0 | 0 |
|  | Mortality^j^ | Total | | 436/3005 | 402/2837 | 838/5842 | NA | | | |
|  |  | Horby and Landray et al | 2022 | 410/2636 | 384/2636 | 794/5272 | 0.336 | 0 | 26 | 1 |
|  |  | Somersan-Karakaya et al | 2022 | 26/369 | 18/201 | 44/570 | 0.416 | 0 | 9 | 0 |
|  | Mortality^k^ | Total | | 1356/7734 | 1513/7428 | 2869/15162 | NA | | | |
|  |  | Horby and Landray et al | 2022 | 943/4839 | 1029/4946 | 1972/9785 | 0.107 | 0 | 14 | 1 |
|  |  | Somersan-Karakaya et al | 2022 | 59/804 | 45/393 | 104/1197 | 0.021 | 4 | 0 | 0 |
|  |  | Weinreich et al | 2020 | 112/736 | 150/748 | 262/1484 | 0.017 | 6 | 0 | 0 |
|  |  | Weinreich et al 2 | 2021 | 242/1355 | 289/1341 | 531/2696 | 0.018 | 9 | 0 | 0 |
|  | Progression to present clinical symptoms^i^ | Total | | 99/942 | 57/945 | 156/1887 | NA | | | |
|  |  | Herman et al | 2022 | 70/842 | 13/841 | 83/1683 | <0.001 | 36 | 0 | 1 |
|  |  | O'Brien et al | 2022 | 29/100 | 44/104 | 73/204 | 0.058 | 0 | 1 | 0 |
|  | Progression to present clinical symptoms^k^ | Total | | 48/884 | 57/396 | 105/1280 | NA | | | |
|  |  | Isa et al | 2022 | 14/729 | 4/240 | 18/969 | 1.000 | 0 | 4 | 1 |
|  |  | O'Brien et al | 2022 | 34/155 | 53/156 | 87/311 | 0.023 | 3 | 0 | 0 |

**(D) Individual study statistics of miscellaneous agents**

| **Treatment** | **Outcome** | **Study Name** | **Study Year** | **Treatment**  **(Event / Population)** | **Control**  **(Event / Population)** | **Total (Event/**  **Population)** | ***P*** | **FI** | **RFI** | **Large** |
| --- | --- | --- | --- | --- | --- | --- | --- | --- | --- | --- |
| Fluvoxamine^13^ | Mortality^l^ | Total | | 86/2352 | 117/2296 | 203/4648 | NA | | | |
|  |  | Lenze et al | 2020 | 0/80 | 4/72 | 4/152 | 0.048 | 1 | 0 | 0 |
|  |  | Reis et al | 2021 | 76/741 | 99/756 | 175/1497 | 0.092 | 0 | 4 | 1 |
|  |  | McCarthy et al | 2023 | 1/670 | 2/607 | 3/1277 | 0.607 | 0 | 1 | 0 |
|  |  | Reiersen et al | 2023 | 8/272 | 10/275 | 18/547 | 0.811 | 0 | 6 | 0 |
|  |  | Stewart et al | 2023 | 1/589 | 2/586 | 3/1175 | 0.624 | 0 | 1 | 0 |
| Colchicine^14^ | Mortality | Total | | 1642/12863 | 1696/12961 | 3338/25824 | NA | | | |
|  |  | Absalon-Aguilar et al | 2021 | 4/56 | 6/60 | 10/116 | 0.744 | 0 | 4 | 1 |
|  |  | Alsultan et al | 2021 | 3/14 | 7/21 | 10/35 | 0.704 | 0 | 3 | 0 |
|  |  | Cecconi et al | 2022 | 7/119 | 10/120 | 17/239 | 0.616 | 0 | 5 | 0 |
|  |  | Deftereos et al | 2020 | 1/55 | 4/50 | 5/105 | 0.189 | 0 | 1 | 0 |
|  |  | Diaz et al | 2021 | 131/640 | 142/639 | 273/1279 | 0.454 | 0 | 18 | 0 |
|  |  | Dorward et al | 2022 | 0/156 | 1/120 | 1/276 | 0.435 | 0 | 0 | 0 |
|  |  | Eikelboom et al | 2022 | 264/1304 | 249/1307 | 513/2611 | 0.460 | 0 | 25 | 1 |
|  |  | Eikelboom et al | 2022 | 12/1939 | 11/1942 | 23/3881 | 0.838 | 0 | 7 | 1 |
|  |  | Gaitan-Duarte et al | 2021 | 22/153 | 28/161 | 50/314 | 0.538 | 0 | 8 | 0 |
|  |  | Gorial et al | 2022 | 1/80 | 3/80 | 4/160 | 0.620 | 0 | 1 | 0 |
|  |  | Kasiri et al | 2023 | 6/55 | 6/51 | 12/106 | 1.000 | 0 | 6 | 0 |
|  |  | Lopes et al | 2021 | 0/36 | 2/36 | 2/72 | 0.493 | 0 | 0 | 0 |
|  |  | Mostafaie et al | 2021 | 1/60 | 6/60 | 7/120 | 0.114 | 0 | 1 | 0 |
|  |  | Pascual-Figal et al | 2021 | 0/52 | 2/51 | 2/103 | 0.243 | 0 | 0 | 0 |
|  |  | Perricone et al | 2022 | 7/77 | 5/75 | 12/152 | 0.765 | 0 | 5 | 0 |
|  |  | Pimenta-Bonifacio et al | 2022 | 0/14 | 2/16 | 2/30 | 0.485 | 0 | 0 | 0 |
|  |  | Rahman et al | 2022 | 4/146 | 13/146 | 17/292 | 0.043 | 1 | 0 | 0 |
|  |  | RECOVERY | 2021 | 1173/5610 | 1190/5730 | 2363/11340 | 0.853 | 0 | 77 | 1 |
|  |  | Sunil et al | 2023 | 1/62 | 0/43 | 1/105 | 1.000 | 0 | 0 | 0 |
|  |  | Tardif et al | 2021 | 5/2235 | 9/2253 | 14/4488 | 0.423 | 0 | 4 | 1 |
| CCP^15^ | Mortality | Total | | 2548/11303 | 2382/10226 | 4930/21529 | NA | | | |
|  |  | Bajpai et al | 2022 | 3/14 | 1/15 | 4/29 | 0.330 | 0 | 1 | 0 |
|  |  | Rojas et al | 2022 | 6/46 | 2/45 | 8/91 | 0.267 | 0 | 2 | 0 |
|  |  | Song et al | 2022 | 22/87 | 7/42 | 29/129 | 0.369 | 0 | 3 | 0 |
|  |  | Jalili et al | 2022 | 16/60 | 11/60 | 27/120 | 0.382 | 0 | 5 | 0 |
|  |  | Thorlacius-Ussing et al | 2022 | 7/99 | 2/46 | 9/145 | 0.719 | 0 | 2 | 0 |
|  |  | Devos et al | 2022 | 20/257 | 7/135 | 27/392 | 0.405 | 0 | 4 | 1 |
|  |  | Sekine et al | 2022 | 18/80 | 13/80 | 31/160 | 0.424 | 0 | 6 | 0 |
|  |  | Kirenga et al | 2021 | 10/69 | 8/67 | 18/136 | 0.801 | 0 | 6 | 0 |
|  |  | Begin et al | 2021 | 156/625 | 69/313 | 225/938 | 0.332 | 0 | 9 | 1 |
|  |  | Bajpai et al 2 | 2022 | 42/200 | 37/200 | 79/400 | 0.616 | 0 | 11 | 0 |
|  |  | Self et al | 2022 | 89/482 | 80/465 | 169/947 | 0.671 | 0 | 17 | 1 |
|  |  | Agarwal et al | 2020 | 34/235 | 31/229 | 65/464 | 0.791 | 0 | 12 | 1 |
|  |  | Gonzalez et al | 2021 | 70/130 | 32/60 | 102/190 | 1.000 | 0 | 10 | 0 |
|  |  | RECOVERY | 2021 | 1399/5795 | 1408/5763 | 2807/11558 | 0.729 | 0 | 74 | 1 |
|  |  | REMAP-CAP | 2021 | 401/1075 | 347/904 | 748/1979 | 0.642 | 0 | 35 | 1 |
|  |  | Simonovich et al | 2021 | 25/228 | 12/105 | 37/333 | 1.000 | 0 | 14 | 1 |
|  |  | Baldeon et al | 2022 | 7/63 | 12/95 | 19/158 | 1.000 | 0 | 5 | 0 |
|  |  | De Santis et al | 2022 | 11/36 | 25/71 | 36/107 | 0.671 | 0 | 6 | 0 |
|  |  | Ortigoza et al | 2022 | 59/462 | 71/462 | 130/924 | 0.298 | 0 | 10 | 1 |
|  |  | van den Berg et al | 2022 | 11/52 | 13/51 | 24/103 | 0.647 | 0 | 6 | 0 |
|  |  | Menichetti et al | 2021 | 14/231 | 19/240 | 33/471 | 0.474 | 0 | 6 | 1 |
|  |  | Bennett-Guerrero et al | 2021 | 16/59 | 5/15 | 21/74 | 0.750 | 0 | 10 | 0 |
|  |  | Bandopadhyay et al | 2021 | 10/40 | 14/40 | 24/80 | 0.465 | 0 | 5 | 0 |
|  |  | Ray et al | 2022 | 10/40 | 14/40 | 24/80 | 0.465 | 0 | 5 | 0 |
|  |  | Denkinger et al | 2022 | 11/68 | 16/66 | 27/134 | 0.285 | 0 | 4 | 0 |
|  |  | Li et al | 2020 | 8/51 | 12/50 | 20/101 | 0.327 | 0 | 4 | 0 |
|  |  | Pouladzadeh et al | 2021 | 3/30 | 5/30 | 8/60 | 0.706 | 0 | 3 | 0 |
|  |  | Korper et al | 2021 | 11/53 | 17/52 | 28/105 | 0.191 | 0 | 3 | 0 |
|  |  | Lacombe et al | 2022 | 7/60 | 12/60 | 19/120 | 0.317 | 0 | 4 | 0 |
|  |  | Holm et al | 2021 | 2/17 | 3/14 | 5/31 | 0.636 | 0 | 2 | 0 |
|  |  | AlQahtani et al | 2021 | 1/20 | 2/20 | 3/40 | 1.000 | 0 | 1 | 0 |
|  |  | Gharbharan et al | 2021 | 6/43 | 11/43 | 17/86 | 0.279 | 0 | 3 | 0 |
|  |  | Avendano-Sola et al | 2021 | 7/179 | 14/171 | 21/350 | 0.116 | 0 | 2 | 0 |
|  |  | O'Donnell et al | 2021 | 19/150 | 18/73 | 37/223 | 0.034 | 2 | 0 | 0 |
|  |  | Rasheed et al | 2020 | 1/21 | 8/28 | 9/49 | 0.059 | 0 | 1 | 0 |
|  |  | Ali et al | 2021 | 10/40 | 6/10 | 16/50 | 0.056 | 0 | 1 | 0 |
|  |  | Bar et al | 2021 | 2/40 | 10/39 | 12/79 | 0.013 | 2 | 0 | 0 |
|  |  | FerNAdez-Sanchez et al | 2022 | 4/29 | 8/10 | 12/39 | <0.001 | 8 | 0 | 0 |

**Abbreviations:** P, P-value; FI, Fragility Index; RFI, Reverse Fragility Index; IL, Interleukin; JAK, Janus Kinase; SARS-CoV-2, Severe Acute Respiratory Syndrome Coronavirus-2; CCP, COVID-19 Convalescent Plamsa; NA, Not Applicable.

**Footnotes:** ^a^ Severe patients, ^b^ Non-severe patients, ^c^ Tocilizumab, ^d^ All-cause mortality at day 28, ^e^ All-cause mortality at day 60, ^f^ Sarilumab, ^g^ Anakinra, ^h^ Canakinumab, ^i^ Seronegative baseline patients, ^j^ Seropositive baseline patients, ^k^ Overall baseline patients, ^l^ Inpatients.

**Supplemental Table 2. Comparisons of RoB between large and small trials**

**(A) Comparisons of RoB 1.0 between large and small trials**

|  | **Large (n =13)** | **Small (n=37)** | **P-value** |
| --- | --- | --- | --- |
| **Selection bias^a^** |  |  | 0.101 |
| **Low** | 12 (92) | 37 (100) |  |
| **High** | 0 (0) | 0 (0) |  |
| **Unclear** | 1 (7.69) | 0 (0) |  |
| **Selection bias^b^** |  |  | 0.583 |
| **Low** | 12 (92) | 32 (87) |  |
| **High** | 0 (0) | 1 (2.70) |  |
| **Unclear** | 1 (8) | 4 (11) |  |
| **Performance bias** |  |  | 0.689 |
| **Low** | 7 (54) | 22 (60) |  |
| **High** | 4 (31) | 9 (24) |  |
| **Unclear** | 2 (15) | 6 (16) |  |
| **Detection bias** |  |  | 0.045 |
| **Low** | 11 (85) | 19 (51) |  |
| **High** | 1 (8) | 9 (24) |  |
| **Unclear** | 1 (8) | 9 (24) |  |
| **Attrition bias** |  |  | 0.307 |
| **Low** | 13 (100) | 34 (91.89) |  |
| **High** | 0 (0) | 2 (5.41) |  |
| **Some concerns** | 0 (0) | 1 (2.7) |  |
| **Reporting bias** |  |  | 0.307 |
| **Low** | 13 (100) | 34 (92) |  |
| **High** | 0 (0) | 0 (0) |  |
| **Unclear** | 0 (0) | 3 (8) |  |
| **Other bias^c^** |  |  | 0.055 |
| **Low** | 11 (85) | 20 (54) |  |
| **High** | 0 (0) | 0 (0) |  |
| **Unclear** | 2 (14) | 17 (46) |  |

**(B) Comparisons of RoB 2.0 between large and small trials**

|  | **Large (n=33)** | **Small (n=138)** | **P-value** |
| --- | --- | --- | --- |
| **D1^d^** |  |  | 0.186 |
| **Low** | 27 (82) | 98 (71) |  |
| **High** | 0 (0) | 5 (4) |  |
| **Some concerns** | 6 (18) | 35 (25) |  |
| **D2^e^** |  |  | 0.267 |
| **Low** | 23 (70) | 84 (61) |  |
| **High** | 0 (0) | 8 (6) |  |
| **Some concerns** | 10 (30) | 46 (33) |  |
| **D3^f^** |  |  | 0.031 |
| **Low** | 32 (97) | 113 (82) |  |
| **High** | 0 (0) | 2 (1) |  |
| **Some concerns** | 1 (3) | 23 (17) |  |
| **D4^g^** |  |  | 0.048 |
| **Low** | 33 (100) | 123 (89) |  |
| **High** | 0 (0) | 0 (0) |  |
| **Some concerns** | 0 (0) | 15 (11) |  |
| **D5^h^** |  |  | 0.226 |
| **Low** | 31 (94) | 119 (86) |  |
| **High** | 0 (0) | 1 (1) |  |
| **Some concerns** | 2 (6) | 18 (13) |  |
| **Overall** |  |  | 0.283 |
| **Low** | 17 (52) | 61 (44) |  |
| **High** | 1 (3) | 15 (11) |  |
| **Some concerns** | 15 (45) | 62 (45) |  |

**Abbreviations:** RoB, Risk of Bias; NA, Not Applicable.

**Footnotes:** ^a^ Random sequence generation, ^b^ Allocation concealment, ^c^ Other biases include funding bias, early stopping bias, baseline imbalance, contamination bias, co-intervention bias, etc, ^d^ Bias arising from the randomization process, ^e^ Bias due to deviations from intended intervention, ^f^ Bias due to missing outcome data, ^g^ Bias in measurement of the outcome, ^h^ Bias in selection of the reported result.

**Supplemental Table 3. Individual study RoB evaluation**

**(A) Studies evaluated by RoB 1.0 tool**

| **Treatment** | **Outcome** | **Study Name** | **Study Year** | **Selection bias^a^** | | **Selection bias^b^** | | **Perform-ance bias** | | **Detection bias** | | **Attrition bias** | | **Reporting bias** | | **Other bias^c^** |
| --- | --- | --- | --- | --- | --- | --- | --- | --- | --- | --- | --- | --- | --- | --- | --- | --- |
| Remdesivir^1^ | Mortality | NA | | | | | | | | | | | | | | |
|  |  | Christoph D. Spinner et al | 2020 | Low | | Low | | High | | Low | | Low | | Low | | Low |
|  |  | Florence Ader et al | 2022 | Low | | Low | | High | | High | | Low | | Low | | Low |
|  |  | J.H. Beigel et al | 2020 | Low | | Low | | Low | | Unclear | | Low | | Low | | Low |
|  |  | Lakshmi Mahajan et al | 2021 | Low | | Low | | High | | High | | Low | | Low | | Unclear |
|  |  | Sherief Abd-Elsalam et al | 2022 | Low | | Low | | High | | Low | | Low | | Low | | Unclear |
|  |  | WHO Solidarity Trial Consortium | 2021 | Low | | Low | | Unclear | | Unclear | | Low | | Low | | Low |
|  |  | Yeming Wang et al | 2020 | Low | | Low | | Low | | Unclear | | Unclear | | Low | | Low |
| Cortico-  steroids^7^ | Mortality | NA | | | | | | | | | | | | | | |
|  |  | Angus et al | 2020 | Low | | Unclear | | High | | High | | Low | | Low | | Unclear |
|  |  | Clemency et al | 2022 | Low | | Low | | Low | | Unclear | | Low | | Unclear | | Low |
|  |  | Dequin et al | 2020 | Low | | Low | | Low | | Unclear | | Low | | Low | | Unclear |
|  |  | Edalatifard et al | 2020 | Low | | Unclear | | Low | | High | | Low | | Low | | Low |
|  |  | Ezer et al | 2021 | Low | | Low | | Low | | High | | Low | | Unclear | | Unclear |
|  |  | Ghanel et al | 2021 | Low | | Low | | High | | High | | Low | | Low | | Unclear |
|  |  | Jamaati et al | 2021 | Low | | Unclear | | Unclear | | Unclear | | Low | | Low | | Unclear |
|  |  | Jeronimo et al | 2021 | Low | | Unclear | | Low | | Unclear | | Low | | Low | | Low |
|  |  | Luis Corral-Gudino et al | 2021 | Low | | Low | | High | | High | | Low | | Low | | Unclear |
|  |  | Marie et al | 2021 | Low | | Low | | Low | | Low | | Low | | Low | | Low |
|  |  | RECOVERY Collaborative Group | 2021 | Low | | Low | | High | | High | | Low | | Low | | Low |
|  |  | Tang et al | 2021 | Low | | Low | | Low | | Low | | Low | | Low | | Unclear |
|  |  | Tomazini et al | 2020 | Low | | Low | | High | | High | | Low | | Low | | Unclear |
|  |  | Yu et al | 2021 | Low | | Low | | High | | High | | Low | | Low | | Unclear |
| JAK Inhibitors^9^ | Mortality | NA | | | | | | | | | | | | | | |
|  |  | Bari/EU-SolidAct | 2022 | Low | | Low | | Low | | Low | | Low | | Low | | Low |
|  |  | Kalil et al | 2021 | Low | | Low | | Low | | Low | | Low | | Low | | Low |
|  |  | Marconi et al | 2021 | Low | | Low | | Low | | Low | | Low | | Low | | Low |
|  |  | Wesley-Ely et al | 2021 | Low | | Low | | Low | | Low | | Low | | Low | | Low |
|  |  | Wolfe et al | 2022 | Low | | Low | | Low | | Low | | Low | | Low | | Low |
| Interferon^10^ | Mortality^d^ | NA | | | | | | | | | | | | | | |
|  |  | Ader et al | 2021 | | Low | | Low | | Unclear | | Unclear | | Low | | Low | Unclear |
|  |  | Alavi Darazam et al | 2021 | | Low | | Low | | Unclear | | Low | | Low | | Low | Unclear |
|  |  | Davoudi-Monfared et al | 2020 | | Low | | Low | | Unclear | | Unclear | | High | | Low | Unclear |
|  |  | Kalil et al | 2021 | | Low | | Low | | Low | | Low | | Low | | Low | Unclear |
|  |  | Pan et al | 2021 | | Unclear | | Unclear | | Unclear | | Low | | Low | | Low | Unclear |
|  |  | Pandit et al | 2021 | | Low | | High | | Unclear | | Unclear | | Low | | Low | Unclear |
|  |  | Rahmani et al | 2020 | | Low | | Low | | Unclear | | Low | | High | | Low | Unclear |
| Anti-SARS-CoV-2 monoclonal antibodies^12^ | Mortality^e^ | NA | | | | | | | | | | | | | | |
|  |  | Horby and Landray et al | 2022 | Low | | Low | | High | | Low | | Low | | Low | | Low |
|  |  | Somersan-Karakaya et al | 2022 | Low | | Low | | Low | | Low | | Low | | Low | | Low |
|  | Mortality^f^ | NA | | | | | | | | | | | | | | |
|  |  | Horby and Landray et al | 2022 | Low | | Low | | High | | Low | | Low | | Low | | Low |
|  |  | Somersan-Karakaya et al | 2022 | Low | | Low | | Low | | Low | | Low | | Low | | Low |
|  | Mortality^g^ | NA | | | | | | | | | | | | | | |
|  |  | Horby and Landray et al | 2022 | Low | | Low | | High | | Low | | Low | | Low | | Low |
|  |  | Somersan-Karakaya et al | 2022 | Low | | Low | | Low | | Low | | Low | | Low | | Low |
|  |  | Weinreich et al | 2020 | Low | | Low | | Low | | Low | | Low | | Low | | Low |
|  |  | Weinreich et al 2 | 2021 | Low | | Low | | Low | | Low | | Low | | Low | | Low |
|  | Progression to present clinical symptoms^e^ | NA | | | | | | | | | | | | | | |
|  |  | Herman et al | 2022 | Low | | Low | | Low | | Low | | Low | | Low | | Low |
|  |  | O'Brien et al | 2022 | Low | | Low | | Low | | Low | | Low | | Low | | Low |
|  | Progression to present clinical symptoms^g^ | NA | | | | | | | | | | | | | | |
|  |  | Isa et al | 2022 | Low | | Low | | Low | | Low | | Low | | Low | | Low |
|  |  | O'Brien et al | 2022 | Low | | Low | | Low | | Low | | Low | | Low | | Low |
| Fluvoxamine^13^ | Mortality^h^ | NA | | | | | | | | | | | | | | |
|  |  | Lenze et al | 2020 | Low | | Low | | Low | | Low | | Low | | Low | | Low |
|  |  | Reis et al | 2021 | Low | | Low | | Low | | Low | | Low | | Low | | Low |
|  |  | McCarthy et al | 2023 | Low | | Low | | Low | | Low | | Low | | Low | | Low |
|  |  | Reiersen et al | 2023 | Low | | Low | | Low | | Low | | Low | | Unclear | | Unclear |
|  |  | Stewart et al | 2023 | Low | | Low | | Low | | Low | | Low | | Low | | Low |

**(B) Studies evaluated by RoB 2.0 tool**

| **Treatment** | **Outcome** | **Study Name** | **Study Year** | **D1^i^** | **D2^j^** | **D3^k^** | **D4^l^** | **D5^m^** | **Overall** |
| --- | --- | --- | --- | --- | --- | --- | --- | --- | --- |
| Molnupiravir^2^ | Mortality | NA | | | | | | | |
|  |  | Arribas et al | 2021 | Some concerns | Some concerns | Low | Some concerns | Some concerns | Low |
|  |  | Butler et al | 2022 | Some concerns | Low | Low | Low | Some concerns | High |
|  |  | Caraco et al | 2022 | Some concerns | Some concerns | Some concerns | Some concerns | Some concerns | Some concerns |
|  |  | Fischer II et al | 2022 | Some concerns | Some concerns | Some concerns | Some concerns | Some concerns | Some concerns |
|  |  | Bernal et al | 2022 | Some concerns | Some concerns | Some concerns | Some concerns | Some concerns | Some concerns |
|  |  | Khoo et al | 2022 | Some concerns | Some concerns | Some concerns | Some concerns | Some concerns | Some concerns |
|  |  | Sinha et al | 2022 | Some concerns | Low | Low | Some concerns | Some concerns | Low |
|  |  | Tippabhotla et al | 2022 | Some concerns | Low | Some concerns | Some concerns | Some concerns | Low |
|  |  | Zou et al | 2022 | Some concerns | Low | Some concerns | Low | Some concerns | Low |
| Ivermectin^3^ | Mortality | NA | | | | | | | |
|  |  | Bounfrate et al | 2021 | Low | Low | Low | Low | Low | Low |
|  |  | Chaccour et al | 2021 | Low | Low | Low | Low | Low | Low |
|  |  | I-TECH | 2022 | Low | Low | Low | Low | Low | Low |
|  |  | Lopez-Medina et al | 2021 | Low | Some concerns | Low | Low | Low | Some concerns |
|  |  | TOGETHER | 2022 | Low | Low | Low | Low | Low | Low |
|  |  | Vallejos et al | 2021 | Low | Low | Low | Low | Low | Low |
| Lopinavir^4^ | Mortality | NA | | | | | | | |
|  |  | Ader et al | 2021 | Low | Some concerns | Low | Low | Low | Some concerns |
|  |  | Cao et al | 2020 | Low | Some concerns | Low | Low | Low | Some concerns |
|  |  | Horby et al | 2020 | Low | Some concerns | Low | Low | Low | Some concerns |
|  |  | Li et al | 2020 | Low | Some concerns | Low | Low | Low | Some concerns |
|  |  | Pan et al | 2020 | Low | Some concerns | Low | Low | Low | Some concerns |
|  |  | Reis et al | 2020 | Low | Low | Some concerns | Low | Low | Some concerns |
| Hydroxy-chloroquine^4^ | Mortality | NA | | | | | | | |
|  |  | Abd-Elsalam et al | 2020 | Some concerns | Some concerns | Low | Low | Low | Low |
|  |  | Ader et al | 2021 | Low | Some concerns | Low | Low | Low | Low |
|  |  | REMAP-CAP | 2020 | Some concerns | Some concerns | Some concerns | Low | Low | Some concerns |
|  |  | HYDRA | 2020 | Some concerns | Low | Some concerns | Low | Low | Some concerns |
|  |  | CCAP-1 | 2020 | Some concerns | Some concerns | Some concerns | Low | Low | Some concerns |
|  |  | COMHY | 2020 | Some concerns | Low | Some concerns | Low | Low | Some concerns |
|  |  | COV-HCQ | 2020 | Some concerns | Low | Some concerns | Low | Low | Some concerns |
|  |  | CCAP-1 2 | 2020 | Some concerns | Low | Some concerns | Low | Low | Some concerns |
|  |  | OAHU-COVID19 | 2020 | Some concerns | Some concerns | Some concerns | Low | Low | Some concerns |
|  |  | ARCHAIC | 2020 | Some concerns | Some concerns | Some concerns | Low | Low | Some concerns |
|  |  | Cavalcanti et al | 2020 | Low | Some concerns | Low | Low | Low | Some concerns |
|  |  | Chen et al (3) | 2020 | Some concerns | Some concerns | Low | Low | Low | Some concerns |
|  |  | Chen et al (4) | 2020 | Some concerns | Some concerns | High | Low | Low | Some concerns |
|  |  | Chen et al (5) | 2020 | Low | Some concerns | Low | Low | Low | Some concerns |
|  |  | Dubee et al | 2021 | Low | Low | Low | Low | Low | Low |
|  |  | Gonzalez et al | 2021 | Some concerns | Low | Low | Low | Low | Some concerns |
|  |  | Hernadex-Cardenas et al | 2021 | Low | Low | Low | Low | High | High |
|  |  | Horby et al (2) | 2020 | Low | Some concerns | Low | Low | Low | Some concerns |
|  |  | Johnston et al | 2021 | Low | Low | Low | Low | Low | Low |
|  |  | Lyngbakken et al | 2020 | Some concerns | Some concerns | Low | Low | Low | Some concerns |
|  |  | Omrani et al | 2020 | Low | Low | Low | Low | Low | Low |
|  |  | Pan et al | 2020 | Low | Some concerns | Low | Low | Low | Some concerns |
|  |  | PATCH | 2020 | Some concerns | Low | Some concerns | Low | Low | Some concerns |
|  |  | TOGETHER | 2020 | Low | Low | Some concerns | Low | Low | Some concerns |
|  |  | Self et al | 2020 | Low | Low | Low | Low | Low | Low |
|  |  | Skipper et al | 2020 | Low | Low | Low | Low | Low | Low |
|  |  | Tang et al | 2020 | Low | Some concerns | Low | Low | Low | Some concerns |
|  |  | Ulrich et al | 2020 | Low | Low | High | Low | Low | High |
| Azithromycin^5^ | Mortality^d^ | NA | | | | | | | |
|  |  | Furtado et al | 2020 | Low | Low | Low | Low | Low | Low |
|  |  | Horby et al | 2021 | Low | Low | Low | Low | Low | Low |
|  | Mortality^n^ | NA | | | | | | | |
|  |  | Cavalcanti et al | 2020 | Low | Low | Low | Low | Low | Low |
|  |  | Sekhavati et al | 2020 | Low | Low | Low | Low | Some concerns | High |
|  |  | Hinks et al | 2021 | Low | Low | Low | Low | Low | Low |
| Favipiravir^6^ | Mortality | NA | | | | | | | |
|  |  | AlQahtani et al | 2022 | Low | Low | Low | Low | Low | Low |
|  |  | Chuah et al | 2022 | Low | Some concerns | Low | Low | Low | Some concerns |
|  |  | Finberg et al | 2021 | Some concerns | Low | Low | Low | Low | Some concerns |
|  |  | Ivashchenko et al | 2021 | Some concerns | Low | Low | Low | Low | Some concerns |
|  |  | Pushkar et al | 2020 | Some concerns | Low | Low | Low | Low | Some concerns |
|  |  | Shah et al | 2023 | Low | Low | Low | Low | Low | Low |
|  |  | Shenoy et al | 2021 | Some concerns | Low | Low | Low | Low | Some concerns |
|  |  | Shinkai et al | 2021 | Some concerns | Low | Low | Low | Low | Some concerns |
|  |  | Udwadia et al | 2021 | Low | Low | Low | Low | Low | Low |
|  |  | Zhao et al | 2021 | Low | Low | Low | Low | Low | Low |
| IL-6 Receptor Blockers^8^ | Mortality^o,p^ | NA | | | | | | | |
|  |  | Declercq J et al | 2021 | Low | Low | Low | Low | Low | Low |
|  |  | IMMCOVA | 2021 | Low | Low | Low | Low | Low | Low |
|  |  | HMO-0224-20 | 2021 | High | Low | Low | Low | Low | High |
|  |  | COVITOZ-01 | 2021 | Low | Low | Low | Low | Low | Low |
|  |  | Broman N et al | 2022 | Low | Some concerns | Low | Low | Some concerns | Some concerns |
|  |  | COVIDOSE-2 | 2021 | Low | Low | Low | Low | Low | Low |
|  |  | Hermine O et al | 2022 | Low | Low | Some concerns | Low | Low | Some concerns |
|  |  | ARCHITECTS | 2021 | Low | Low | Low | Low | Low | Low |
|  |  | Talaschian M et al | 2021 | Some concerns | Some concerns | Some concerns | Low | Some concerns | High |
|  |  | Rutgers A et al | 2021 | Some concerns | Low | Low | Low | Low | Some concerns |
|  |  | Soin AS et al | 2021 | Low | Some concerns | Low | Low | Low | Some concerns |
|  |  | Horby P et al | 2021 | Low | Low | Low | Low | Low | Low |
|  |  | Veiga VC et al | 2021 | Low | Some concerns | Low | Low | Low | Some concerns |
|  |  | Gordon AC et al | 2021 | Low | Some concerns | Low | Low | Low | Some concerns |
|  |  | Salama C et al | 2020 | Low | Low | Low | Low | Low | Low |
|  |  | Stone JH et al | 2020 | Low | Low | Low | Low | Low | Low |
|  |  | Salvarani C et al | 2020 | Low | Some concerns | Low | Low | Low | Some concerns |
|  |  | Hermine O et al | 2020 | Low | Some concerns | Low | Low | Low | Some concerns |
|  |  | Rosas IO et al | 2022 | Low | Low | Low | Low | Low | Low |
|  | Mortality^o,q^ | NA | | | | | | | |
|  |  | Declercq J et al | 2021 | Low | Low | Low | Low | Low | Low |
|  |  | HMO-0224-20 | 2021 | High | Low | Low | Low | Low | High |
|  |  | COVITOZ-01 | 2021 | Low | Low | Low | Low | Low | Low |
|  |  | Broman N et al | 2022 | Low | Low | Low | Low | Low | Low |
|  |  | Hermine O et al | 2022 | Low | Low | Some concerns | Low | Low | Some concerns |
|  |  | ARCHITECTS | 2021 | Low | Low | Low | Low | Low | Low |
|  |  | Derde L et al | 2021 | Low | Some concerns | Low | Low | Low | Some concerns |
|  |  | Salama C et al | 2020 | Low | Low | Low | Low | Low | Low |
|  |  | Hermine O et al | 2020 | Low | Some concerns | Low | Low | Low | Some concerns |
|  |  | Rosas IO et al | 2022 | Low | Low | Low | Low | Low | Low |
|  | Clinical improve-ment^r^ | NA | | | | | | | |
|  |  | Branch-Elliman W et al | 2022 | Low | Low | Low | Some concerns | Some concerns | Some concerns |
|  |  | Merchante N et al | 2021 | Low | Some concerns | Low | Some concerns | Low | Some concerns |
|  |  | Garcia-Vicuna R et al | 2022 | Low | Some concerns | Low | Some concerns | Low | Some concerns |
|  |  | Sancho-Lopez A et al | 2021 | Low | Some concerns | Low | Some concerns | Low | Some concerns |
|  |  | Hermine O et al | 2022 | Low | Low | Some concerns | Some concerns | Some concerns | Some concerns |
|  |  | Mariette X et al | 2021 | Low | Some concerns | Low | Some concerns | Some concerns | Some concerns |
|  |  | Sivapalasingam et al | 2022 | Some concerns | Low | Some concerns | Low | Some concerns | Some concerns |
|  |  | Sivapalasingam et al 2 | 2022 | Some concerns | Low | Some concerns | Low | Some concerns | Some concerns |
| IL-1 Receptor Blockers^11^ | Mortality^s^ | NA | | | | | | | |
|  |  | Audemard-Verger et al | 2022 | Low | Low | Low | Low | Low | Low |
|  |  | CORIMUNO-19 Collaborative group | 2021 | Low | High | Low | Low | Low | High |
|  |  | Declercq et al | 2021 | Low | High | Low | Low | Low | High |
|  |  | Kharazmi et al | 2021 | High | High | Low | Low | Low | High |
|  |  | Kyriazopoulou et al | 2021 | Low | Low | Low | Low | Low | Low |
|  | Mortality^t^ | NA | | | | | | | |
|  |  | Caricchio et al | 2021 | Low | Low | Low | Low | Low | Low |
|  |  | NCT04365153 | 2022 | High | Low | Low | Low | Low | High |
|  | Risk of requiring mechanical ventilation | NA | | | | | | | |
|  |  | Audemard-Verger et al | 2022 | Low | Low | Low | Low | Low | Low |
|  |  | Caricchio et al | 2021 | Low | Low | Low | Low | Low | Low |
|  |  | CORIMUNO-19 Collaborative group | 2021 | Low | High | Low | Low | Low | High |
|  |  | Kharazmi et al | 2021 | High | High | Low | Low | Low | High |
|  |  | Kyriazopoulou et al | 2021 | Low | Low | Low | Low | Low | Low |
| Colchicine^14^ | Mortality | NA | | | | | | | |
|  |  | Absalon-Aguilar et al | 2021 | Low | Low | Low | Low | Low | Low |
|  |  | Alsultan et al | 2021 | Some concerns | Some concerns | Low | Low | Low | Some concerns |
|  |  | Cecconi et al | 2022 | Low | Low | Low | Low | Low | Low |
|  |  | Deftereos et al | 2020 | Low | Low | Low | Low | Low | Low |
|  |  | Diaz et al | 2021 | Low | Low | Low | Low | Low | Low |
|  |  | Dorward et al | 2022 | Low | High | Low | Some concerns | Low | High |
|  |  | Eikelboom et al | 2022 | Low | Some concerns | Low | Low | Low | Some concerns |
|  |  | Eikelboom et al | 2022 | Some concerns | Some concerns | Low | Low | Low | Some concerns |
|  |  | Gaitan-Duarte et al | 2021 | Low | Low | Low | Low | Low | Low |
|  |  | Gorial et al | 2022 | Some concerns | Some concerns | Low | Low | Low | Some concerns |
|  |  | Kasiri et al | 2023 | Low | Low | Low | Low | Low | Low |
|  |  | Lopes et al | 2021 | Low | Some concerns | Low | Low | Low | Some concerns |
|  |  | Mostafaie et al | 2021 | Low | Some concerns | Some concerns | Low | Low | Some concerns |
|  |  | Pascual-Figal et al | 2021 | Low | Some concerns | Low | Low | Low | Some concerns |
|  |  | Perricone et al | 2022 | Low | Some concerns | Low | Low | Low | Some concerns |
|  |  | Pimenta-Bonifacio et al | 2022 | Some concerns | Some concerns | Low | Low | Low | Some concerns |
|  |  | Rahman et al | 2022 | Low | Low | Low | Low | Low | Low |
|  |  | RECOVERY | 2021 | Low | Low | Low | Low | Low | Low |
|  |  | Sunil et al | 2023 | Some concerns | Low | Low | Some concerns | Some concerns | Some concerns |
|  |  | Tardif et al | 2021 | Low | Low | Low | Low | Low | Low |
| CCP^15^ | Mortality | NA | | | | | | | |
|  |  | Bajpai et al | 2022 | Low | Low | Low | Low | Low | Low |
|  |  | Rojas et al | 2022 | Some concerns | Some concerns | Low | Low | Low | Some concerns |
|  |  | Song et al | 2022 | Low | Low | Low | Low | Low | Low |
|  |  | Jalili et al | 2022 | Low | Low | Low | Low | Low | Low |
|  |  | Thorlacius-Ussing et al | 2022 | Low | Low | Low | Low | Low | Low |
|  |  | Devos et al | 2022 | Low | Low | Low | Low | Low | Low |
|  |  | Sekine et al | 2022 | Low | Low | Low | Low | Low | Low |
|  |  | Kirenga et al | 2021 | Low | Low | Low | Low | Low | Low |
|  |  | Begin et al | 2021 | Low | Some concerns | Low | Low | Low | Some concerns |
|  |  | Bajpai et al 2 | 2022 | Low | Low | Low | Low | Low | Low |
|  |  | Self et al | 2022 | Low | Low | Low | Low | Low | Low |
|  |  | Agarwal et al | 2020 | Low | Some concerns | Low | Low | Low | Some concerns |
|  |  | Gonzalez et al | 2021 | Low | Low | Low | Low | Low | Low |
|  |  | RECOVERY | 2021 | Low | Low | Low | Low | Low | Some concerns |
|  |  | REMAP-CAP | 2021 | Low | Low | Low | Low | Low | Low |
|  |  | Simonovich et al | 2021 | Low | Low | Low | Low | Low | Low |
|  |  | Baldeon et al | 2022 | Low | Low | Low | Low | Low | Low |
|  |  | De Santis et al | 2022 | Low | Low | Low | Low | Low | Low |
|  |  | Ortigoza et al | 2022 | Low | Low | Low | Low | Low | Low |
|  |  | van den Berg et al | 2022 | Low | Low | Low | Low | Low | Low |
|  |  | Menichetti et al | 2021 | Low | Low | Low | Low | Low | Low |
|  |  | Bennett-Guerrero et al | 2021 | Low | Low | Low | Low | Low | Low |
|  |  | Bandopadhyay et al | 2021 | Some concerns | Low | Low | Low | Low | Some concerns |
|  |  | Ray et al | 2022 | Low | Some concerns | Low | Low | Some concerns | Some concerns |
|  |  | Denkinger et al | 2022 | Low | High | Low | Low | Low | High |
|  |  | Li et al | 2020 | Low | Some concerns | Low | Low | Low | Some concerns |
|  |  | Pouladzadeh et al | 2021 | Low | Low | Low | Low | Low | Low |
|  |  | Korper et al | 2021 | Low | High | Low | Low | Low | High |
|  |  | Lacombe et al | 2022 | Low | Low | Low | Low | Low | Low |
|  |  | Holm et al | 2021 | Some concerns | Some concerns | Low | Low | Low | Some concerns |
|  |  | AlQahtani et al | 2021 | Low | Low | Low | Low | Some concerns | Some concerns |
|  |  | Gharbharan et al | 2021 | Low | Some concerns | Low | Low | Low | Some concerns |
|  |  | Avendano-Sola et al | 2021 | Low | Some concerns | Low | Low | Low | Some concerns |
|  |  | O'Donnell et al | 2021 | Low | Low | Low | Low | Low | Low |
|  |  | Rasheed et al | 2020 | Low | Low | Low | Low | Low | Low |
|  |  | Ali et al | 2021 | Low | Low | Low | Low | Low | Low |
|  |  | Bar et al | 2021 | Low | Low | Low | Low | Low | Low |
|  |  | FerNAdez-Sanchez et al | 2022 | Some concerns | Low | Low | Low | Low | Some concerns |

**Abbreviations:** RoB, Risk of Bias; IL, Interleukin; JAK, Janus Kinase; SARS-CoV-2, Severe Acute Respiratory Syndrome Coronavirus-2; CCP, COVID-19 Convalescent Plasma; NA, Not Applicable (For visually separating meta-analyses).

**Footnotes:** ^a^ Random sequence generation, ^b^ Allocation concealment, ^c^ Other biases include funding bias, early stopping bias, baseline imbalance, contamination bias, co-intervention bias, etc, ^d^ Severe, ^e^ Seronegative baseline patients, ^f^ Seropositive baseline patients, ^g^ Overall baseline patients, ^h^ Inpatients, ^i^ Bias arising from the randomization process, ^j^ Bias due to deviations from intended intervention, ^k^ Bias due to missing outcome data, ^l^ Bias in measurement of the outcome, ^m^ Bias in selection of the reported result, ^n^ Non-sever patients, ^o^ Tocilizumab, ^p^ All-cause mortality at day 28, ^q^ All-cause mortality at day 60, ^r^ Sarilumab, ^s^ Anakinra, ^t^ Canakinumab.

**Supplemental References**

**1.** Chen C, Fang J, Chen S, et al. The efficacy and safety of remdesivir alone and in combination with other drugs for the treatment of COVID-19: a systematic review and meta-analysis. *BMC infectious diseases.* 2023;23(1):672, <https://doi.org/10.1186/s12879-023-08525-0>.

**2.** Huang P-Y, Liu T-H, Wu J-Y, Tsai Y-W, Lai C-C. Clinical efficacy and safety of molnupiravir for nonhospitalized and hospitalized patients with COVID-19: A systematic review and meta-analysis of randomized control trials. *Journal of Medical Virology.* 2023;95(3):e28621, <https://doi.org/10.1002/jmv.28621>.

**3.** Popp M, Stegemann M, Metzendorf M-I, et al. Ivermectin for preventing and treating COVID-19. *The Cochrane Database of Systematic Reviews.* 2021;7(7):CD015017, <https://doi.org/10.1002/14651858.CD015017.pub2>.

**4.** Moneer O, Daly G, Skydel JJ, et al. Agreement of treatment effects from observational studies and randomized controlled trials evaluating hydroxychloroquine, lopinavir-ritonavir, or dexamethasone for covid-19: meta-epidemiological study. *BMJ.* 2022;377:e069400, <https://doi.org/10.1136/bmj-2021-069400>.

**5.** Kamel AM, Monem MSA, Sharaf NA, Magdy N, Farid SF. Efficacy and safety of azithromycin in Covid-19 patients: A systematic review and meta-analysis of randomized clinical trials. *Reviews in Medical Virology.* 2022;32(1):e2258, <https://doi.org/10.1002/rmv.2258>.

**6.** Naveed A, Cheema HA, Shahid A, et al. Favipiravir for the Treatment of Hospitalized Patients with COVID-19: A Systematic Review and Meta-Analysis of Randomized Controlled Trials. *American Journal of Therapeutics.* 2024, <https://doi.org/10.1097/MJT.0000000000001688>.

**7.** Qiao W, Meng L, Zhang Y, et al. Safety and efficacy of glucocorticoids in the treatment of COVID-19: A meta-analysis of randomized control trials. *Expert Review of Respiratory Medicine.* 2023;17(1):81-96, <https://doi.org/10.1080/17476348.2023.2177155>.

**8.** Ghosn L, Assi R, Evrenoglou T, et al. Interleukin-6 blocking agents for treating COVID-19: a living systematic review. *The Cochrane Database of Systematic Reviews.* 2023;6(6):CD013881, <https://doi.org/10.1002/14651858.CD013881.pub2>.

**9.** Manoharan S, Ying Ying L. Baricitinib statistically significantly reduced COVID-19-related mortality: a systematic review and meta-analysis of five phase III randomized, blinded and placebo-controlled clinical trials. *Biology Methods & Protocols.* 2024;9(1):bpae002, <https://doi.org/10.1093/biomethods/bpae002>.

**10.** Ryoo S, Koh D-H, Yu S-Y, et al. Clinical efficacy and safety of interferon (Type I and Type III) therapy in patients with COVID-19: A systematic review and meta-analysis of randomized controlled trials. *PloS One.* 2023;18(3):e0272826, <https://doi.org/10.1371/journal.pone.0272826>.

**11.** Lan S-H, Hsu C-K, Chang S-P, Lu L-C, Lai C-C. Clinical efficacy and safety of interleukin-1 blockade in the treatment of patients with COVID-19: a systematic review and meta-analysis of randomized controlled trials. *Annals of Medicine.* 2023;55(1):2208872, <https://doi.org/10.1080/07853890.2023.2208872>.

**12.** Wicaksono IA, Suhandi C, Elamin KM, Wathoni N. Efficacy and safety of casirivimab-imdevimab combination on COVID-19 patients: A systematic review and meta-analysis randomized controlled trial. *Heliyon.* 2023;9(12):e22839, <https://doi.org/10.1016/j.heliyon.2023.e22839>.

**13.** Zhou Q, Zhao G, Pan Y, Zhang Y, Ni Y. The efficacy and safety of fluvoxamine in patients with COVID-19: A systematic review and meta-analysis from randomized controlled trials. *PloS One.* 2024;19(5):e0300512, <https://doi.org/10.1371/journal.pone.0300512>.

**14.** Cheema HA, Jafar U, Shahid A, et al. Colchicine for the treatment of patients with COVID-19: an updated systematic review and meta-analysis of randomised controlled trials. *BMJ open.* 2024;14(4):e074373, <https://doi.org/10.1136/bmjopen-2023-074373>.

**15.** Senefeld JW, Gorman EK, Johnson PW, et al. Mortality Rates Among Hospitalized Patients With COVID-19 Treated With Convalescent Plasma: A Systematic Review and Meta-Analysis. *Mayo Clinic Proceedings: Innovations, Quality & Outcomes.* 2023;7(5):499-513, <https://doi.org/10.1016/j.mayocpiqo.2023.09.001>.

**16.** F T, Q F, Z C. Efficacy and Safety of Molnupiravir Treatment for COVID-19: A Systematic Review and Meta-Analysis of Randomized Controlled Trials. *International journal of antimicrobial agents.* 2023;62(2), <https://doi.org/10.1016/j.ijantimicag.2023.106870>.

**17.** Sy Y, M C, C C, et al. Clinical efficacy and safety of SARS-CoV-2-neutralizing monoclonal antibody in patients with COVID-19: A living systematic review and meta-analysis. *Journal of microbiology, immunology, and infection = Wei mian yu gan ran za zhi.* 2023;56(5), <https://doi.org/10.1016/j.jmii.2023.07.009>.

**18.** Feng T, Zhang X, Sun Y, et al. A systematic review and meta‑analysis of Arbidol therapy for acute respiratory viral infections: A potential treatment for COVID‑19. *Experimental and Therapeutic Medicine.* 2022;24(6):736, <https://doi.org/10.3892/etm.2022.11672>.

**19.** Page MJ, McKenzie JE, Bossuyt PM, et al. The PRISMA 2020 statement: an updated guideline for reporting systematic reviews. *BMJ (Clinical research ed.).* 2021;372:n71, <https://doi.org/10.1136/bmj.n71>.
